# Three New Sesquiterpenoids and One New Sesquiterpenoid Derivative from Chinese Eaglewood

**DOI:** 10.3390/molecules21030281

**Published:** 2016-02-27

**Authors:** Huan Zhao, Qinghua Peng, Zhuzhen Han, Li Yang, Zhengtao Wang

**Affiliations:** 1 The MOE Key Laboratory for Standardization of Chinese Medicines and the SATCM Key Laboratory for New Resources and Quality Evaluation of Chinese Medicines, Institute of Traditional Chinese Materia Medica, Shanghai University of Traditional Chinese Medicine, 1200 Cailun Road, Shanghai 201210, China; 12248573@163.com (H.Z.); 15001972558@163.com (Q.P.); ztwang@shutcm.edu.cn (Z.W.); 2 Shanghai R & D Centre for Standardization of Chinese Medicines, Shanghai 201203, China

**Keywords:** Chinese eaglewood, sesquiterpenoids, sesquiterpenoid derivative, X-ray diffraction, anti-inflammatory activity

## Abstract

Three new sesquiterpenoids (**1**–**3**) and one new sesquiterpenoid derivative (**4**), along with three known sesquiterpenoids (**5**–**7**), were isolated from the 95% ethanolic extract of Chinese eaglewood [*Aquilaria sinensis* (Lour.) Gilg]. The structures of these compounds were elucidated through extensive analysis of spectroscopic data including IR, NMR, HRESIMS, and X-ray diffraction experiments. In addition, the above new compounds were detected for their bioactivities against LPS-induced NO production in RAW 264.7 cells. Among them, compound **2** exhibited obvious anti-inflammatory activity with an IC_50_ value of 8.1 μM.

## 1. Introduction

**Figure 1:**
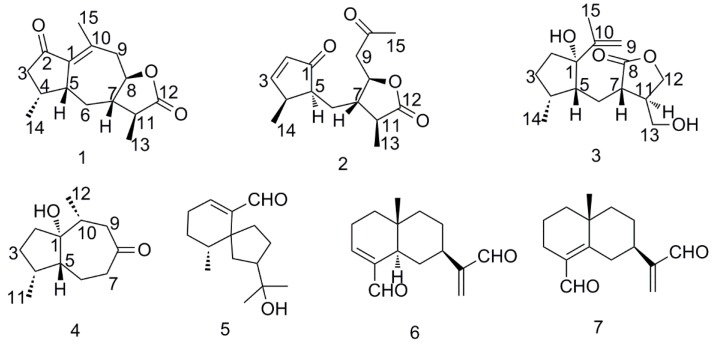
Structures of compounds **1**–**7**.

Chinese eaglewood, the resinous wood of *Aquilaria sinensis* (Lour.) Gilg (Thymelaeaceae), commonly known in different countries as Chenxiang, agarwood, agalloch, jinkoh, or aloeswood, is widely distributed in Southern China in such provinces as Hainan, Fujian, Yunnan and Guangxi [1]. The resinous part of eaglewood can be formed by any of many possible natural or artificial factors, such as lightning strikes, bacteria invasion, insects, burning, holing, physical cutting, and artificial chemical stimulation. Traditionally, eaglewood has been used as analgesic, sedative, and digestive medicine in many Asian countries. Moreover, some rare and precious eaglewoods also serve as incense, items for collection, and decorations all over the world. Research on the chemical components of the eaglewood began half a century ago. So far, a large number of compounds have been reported from this resin wood, including aromatics [2,3], sesquiterpenes [4], chromone derivatives [5,6,7,8], triterpenes, and diterpenes [9]. Some of them showed certain anti-microbial, anti-inflammation, neuroprotective, and anti-depressant activities. Previous phytochemical investigations of Chinese eaglewood have resulted in the isolation and identification of some chromone derivatives and sesquiterpenes [5,10]. As a continuation of studies on the bioactive metabolites from this plant, three new sesquiterpenoids (**1**–**3**) and one new sesquiterpenoid derivative (**4**), together with three known sesquiterpenoids (**5**–**7**) (Figure 1), were isolated from the 95% ethanolic extract of Chinese eaglewood. Their structures were elucidated mainly via IR, NMR, HRESIMS, and X-ray diffraction experiments. Herein, the isolation and structural identification of the compounds obtained from Chinese eaglewood and the inhibitory effects of the new compounds on LPS-induced NO production in RAW 264.7 cells *in vitro* are reported.

## 2. Results and Discussion

### 2.1. Structure Elucidation of Compounds

Compound **1** was obtained as colorless needles via crystallization from acetone. Its molecular formula was established as C_15_H_20_O_3_ on the basis of the [M + Na]^+^ ion peak at *m*/*z* 271.1323 (calcd for C_15_H_20_O_3_Na, 271.1310) in the positive HRESIMS, indicating 6 degrees of unsaturation. The IR spectrum of compound **1** revealed absorptions of carbonyl (1774 cm^−1^), double bond (1614 cm^−1^), and ester group (1706 cm^−1^). Its ^1^H-NMR data (Table 1) suggested the presence of three methyl (δ_H_ 1.21, d, *J* = 6.8 Hz, CH_3_-13; δ_H_ 0.96, d, *J* = 6.8 Hz, CH_3_-14; δ_H_ 2.31, d, *J* = 2.4 Hz, CH_3_-15) and one oxymethine (δ_H_ 3.86, td, *J* = 9.6 Hz, 3.6 Hz) protons. The ^13^C-NMR data (Table 2) displayed 15 carbon signals, sorted into three methyls, three methylenes, five methines, and four quaternary carbons according to DEPT spectrum, respectively. Double-bond carbon (δ_C_ 138.5, C-1; δ_C_ 150.3, C-10), carbonyl (δ_C_ 209.4, C-2), and acetoxyl (δ_C_ 180.7, C-12) signals could also be observed in the ^13^C NMR spectrum. In addition, there were no exchanging hydrogen atoms in compound **1**, based on the molecular formula by combination with the ^13^C-NMR and DEPT spectroscopic data. Therefore, the presence of a tricyclic unit was determined by the distinctive signals at δ_C_ 180.7 and δ_C_ 81.4.

**Figure 2:**
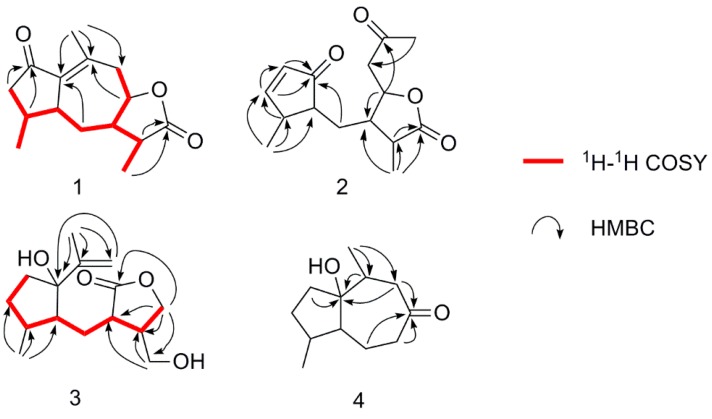
Selected key ^1^H-^1^H COSY and HMBC correlations of compounds **1**–**4**.

In the ^1^H-^1^H COSY experiment, a long spin-system of CH_2_CH(CH_3_) CHCH_2_CH(CHCH_3_)CHCH_2_ [C3/C4(/C14)/C5/C6/C7(/C11/C13)/C8/C9] was deduced by the correlations starting from H-3a and H-3b. Coupled with the HMBC correlations of CH_3_-15 to C-1, C-9, and C-10, correlations of CH_3_-15 to C-12, correlations of H-5 to C-1 and C-2, and correlations of H-4 to C-2, a guaiane-type sesquiterpenoid skeleton was established as shown in Figure 2.

**Figure 3:**
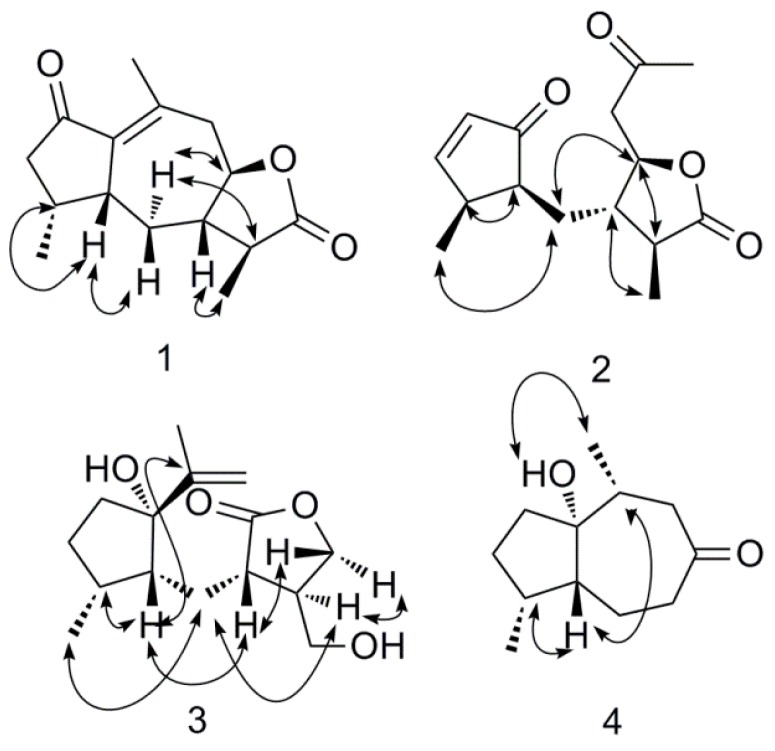
Key NOESY correlations of compounds **1**–**4**.

**Figure 4:**
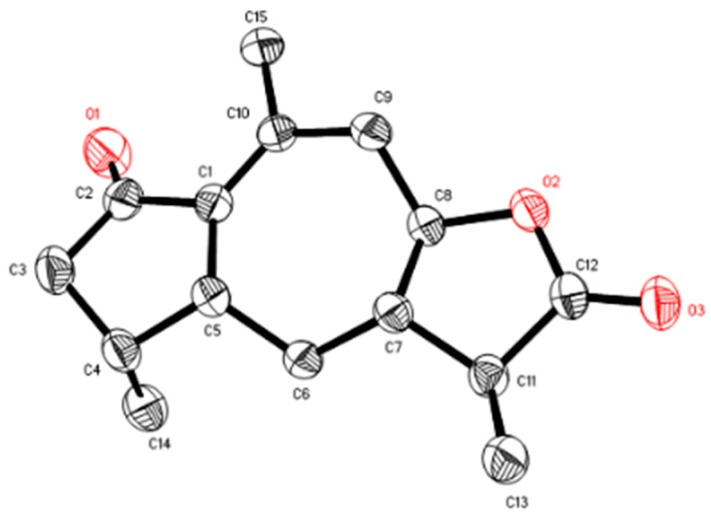
ORTEP diagram of compound **1**.

The α-orientation of Me-14 and H-8 were assigned by the NOESY correlations of H-5/H-4 and H-6a, and H-6b/H-8, whereas, H-5, H-7, and H-11 were determined to be β-oriented through the key correlations of H-6b/ H-11 and Me-11/H-7 (Figure 3). To further affirm the relative configuration of compound **1**, the Cu Kα X-ray diffraction experiment was conducted, which confirmed the above conclusion (Figure 4). Therefore, the structure of compound **1** was identified to be 7βH-guaia-1(10)-en-12,8β-olide (Figure 1).

Compound **2** had the molecular formula C_15_H_20_O_4_, as determined by HRESIMS at *m*/*z* 287.1251 (calcd for C_15_H_20_O_3_Na, 287.1259). The ^1^H-NMR data of compound **2** (Table 1) exhibited three methyl proton signals (δ_H_ 1.30, d, *J* = 7.2 Hz, CH_3_-13; δ_H_ 1.25, d, *J* = 7.2 Hz, CH_3_-14; δ_H_ 2.22, br s, CH_3_-15), an oxygenated methine proton (δ_H_ 4.53, m, H-8), and two olefinic protons (δ_H_ 6.09, dd, *J* = 5.4, 1.8 Hz, H-2; δ_H_ 7.55, dd, *J* = 6.0, 2.4 Hz, H-3). The ^13^C-NMR and DEPT spectra of compound **2** exhibited 15 carbon signals, which was similar to the known compound postiaseco-guaianolide [11,12], except for the differences in the numbers and chemical shifts of double bonds. Detailed analysis of 2D NMR data, the terminal double bond (-11, -13) in postiaseco-guaianolide was substituted by a methyl in compound **2** on the basis of the HMBC correlation of δ_H_ 1.30 to δ_C_ 178.1. Furthermore, the position of the double bond in compound **2** was determined by the key HMBC correlations of H-14 to δ_C_ 168.2 (C-3), and H-2, H-3 to δ_C_ 210.8 (C-1). The NOESY correlation of H-2/H-3 illustrated the geometric configuration of C=C double bond between C-2 and C-3, which belonged to Z-type. The relative configuration of compound **2** was also disclosed through the NOESY spectrum by comparison with postiaseco-guaianolide. On the assumption that H-5 and H-7 were assigned as α-oriented and β-oriented, respectively, the correlations of CH_3_-13/H-7, H-4/H-5, and H-8/H-11 allowed the assignments of H-11, H-4, and H-8 as α-orientation (Figure 3). Consequently, the structure of compound **2** was tentatively identified to be 1,10-dioxo-4αH-5αH-7βH-11αH-1,10-secoguaia-2(3)-en-12,8β-olide (Figure 1).

Compound **3** was obtained as a colorless oil. Its molecular formula was assigned as C_15_H_24_O_4_ by HRESIMS at *m*/*z* 291.1594 (calcd for C_15_H_24_O_4_Na, 291.1572), accounting for four degrees of unsaturation. In the ^1^H-NMR data (Table 1), two methyl groups at δ_H_ (1.06 d, *J* = 7.2 Hz, CH_3_-14 and 1.81 s, CH_3_-15), and two olefinic protons at δ_H_ (4.90 s, 5.07 s) were observed. The ^13^C-NMR and DEPT spectra showed 15 carbon signals including two methyls, six methylenes (an oxygenated and one olefinic carbons), four methines, and three quaternary carbons (one carbonyl, an oxygenated, and one olefinic carbons). The ^1^H-^1^H COSY, HSQC, and HMBC spectra disclosed that compound **3** was an 8, 9 secoguaiane-type sesquiterpenoid (Figure 2). The ^1^H-^1^H COSY correlations of H-7/H-11, H-11/H-12 and the HMBC correlation of H-12 to δ_C_ 210.8 (C-8) attested to the presence of a five-numbered lactone ring. The HMBC correlations of H-15 and H-9 to δ_C_ 85.8 (C-1) determined that the isopropenyl moiety was connected to C-1. Combined with the ^1^H-^1^H COSY correlations of H-2 through H-3, H-4, H-5, H-6 to H-7, HMBC correlations of Me-14 to C-3, C-4, and C-5, and HMBC correlations of H-13 to C-11 and C-12, the planar structure of compound **3** was deduced as shown in Figure 1. The relative configuration was inferred from the NOESY spectrum in reference to compound **1**. If H-5 and H-7 were both assigned to be β-oriented, H-11 and Me-14 were situated at the opposite side of the molecule with α-orientation by NOESY correlations of H-13/H-7 and H-4/H-5. The 1-OH group was inferred to be α-oriented according to the NOESY signal between H-4 and Me-15. Therefore, the structure of compound **3** was tentatively established to be 1α-hydroxy-4βH-5βH-7βH-11αH-8,9-secoguaia-9(10)-en-8,12-olide (Figure 1).

Compound **4** was isolated as a colorless oil. Its molecular formula was assigned as C_12_H_20_O_2_ on the basis of the [M + Na]^+^ ion peak at *m*/*z* 219.1387 (calcd for C_12_H_20_O_2_Na, 219.1361) in the positive HRESIMS. The IR spectrum of compound **4** showed absorptions for hydroxyl (3447 cm^−1^) and carbonyl (1698 cm^−1^) groups. The ^1^H-NMR data (Table 1) displayed two methyl groups at δ_H_ (0.98 d, *J* = 7.2 Hz, H_3_-11 and 1.08 d, *J* = 6.8 Hz, CH_3_-12). The ^13^C-NMR and DEPT spectra (Table 2) exhibited 12 carbon signals including two methyls, five methylenes, three methines, and two quaternary carbons (one carbonyl and an oxygenated carbons). The above information indicated that compound **4** was identified as a natural sesquiterpenoid derivative. The interpretation of 1D and 2D NMR data disclosed that compound **4** originated from guaianesesquiterpenoid by degradation of an isopropyl fragment (C-11/12/13). The positions of the hydroxyl group (δ_C_ 86.9, qC) and the carbonyl group (δ_C_ 213.9) were deduced via HMBC correlations (Figure 2). The key NOESY correlations of H-4/H-5, H-10/H-5 were observed, which disclosed that Me-11 and Me-12 were α-oriented while H-5 was β-oriented. The relative configuration of OH-1 was determined by the NOESY correlation of between OH-1 (δ_H_ 4.13, in DMSO-d_6_) and Me-12 (Figure 3). Thus, the structure of compound **4** was elucidated to be 1α-hydroxy-4α,10α-dimethyl-5βH-octahydro-azulen-8-one (Figure 1).

Through comparing their spectroscopic data with the literature, compound **3** known compounds were identified as baimuxinal (**5**) [13], Selina-3,11-diene-12,15-dial (**6**) [14], and Selina-4,11-diene-12,15-dial (**7**) [15], respectively.

### 2.2. Evalution of Anti-Inflammatory Activity

Compounds **1**–**4** were tested for their inhibitory effects against LPS induced NO production in RAW 264.7 cells. Aminoguanidine hydrochloride acted as the positive control. The results showed that compound **2** exhibited significant inhibitory activity with an IC_50_ value of 8.1 μM, compared to the positive control Aminoguanidine hydrochloride with an IC_50_ value of 11.6 μM, while compounds **1**, **3**, and **4** did not exhibit obvious inhibitory activities with IC_50_ values higher than 100 μM. In addition, none of the compounds showed apparent cytotoxicities (see Supplementary Materials).

## 3. Experimental Section

### 3.1. General Experimental Procedures

Optical rotations were measured on a Krüss-P800-T polarimeter (A. Krüss Optronic, Hamburg, Germany). IR spectra were recorded on a JASCO J-180 spectrometer (Jasco, Hachioji, Japan). Melting point was carried out with a microscopic melting point meter (WRX-4) (Shanghai Yice instrument Co., Ltd., Shanghai, China). NMR spectra were run on a Bruker AVANCE-Ш instrument (Bruker, Rheinstetten, Germany) operating at 600 MHz or 400 MHz with tetramethylsilane (TMS) as internal standard. HRESIMS spectra were obtained on a Waters UPLC Premier QTOF spectrometer (Waters Corp., Milford, MA, USA). Column chromatography was performed with silica gel (200–300 mesh, Qingdao Marine Chemical Inc., Qingdao, China), Sephadex LH-20 (GE Healthcare Bio-Sciences AB, Uppsala, Sweden), and YMC gel ODS-A-HG (50 μm, YMC Co., Ltd., Kyoto, Japan). Thin-layer chromatography (TLC) analysis was run on HSGF_254_ plates (YantaiJiangyou Silica Gel Development Co., Ltd., Yantai, China), spraying with 5% vanillin-sulfuric acid. Reversed phase medium pressure liquid chromatography (RP-MPLC) was performed on a Tong Heng Innovation Sepacore system (Beijing Chuangxintongheng Science & Technology Co., Ltd., Beijing, China). Preparative TLC was carried out with glass precoated silica gel HSGF_254_ (YantaiJiangyou Silica Gel Development Co., Ltd., Yantai, China).

### 3.2. Plant Material

Chinese eaglewood was collected from an herbal medicine market in Bozhou, Anhui Province in July 2014 and was authenticated by professor Li-Hong Wu. A voucher specimen (No. CX140724) was deposited in the herbarium of the Institute of Chinese Materia Medica, Shanghai University of Traditional Chinese Medicine.

### 3.3. Extraction and Isolation

Chinese eaglewood (1.78 kg) was crushed and soaked firstly, then extracted with 95% ethanol (3 × 20 L, 2 h each time) by reflux. The extracts were concentrated to yield a residue (146.5 g) under reduced pressure, which was chromatographed on a silica gel column (1.5 kg, 200–300 mesh, 80 × 10 cm), eluted with a step gradient of petroleum ether-EtOAc (100:0, 50:1, 10:1, 5:1, 3:1, 1:1, 0:100, *v*/*v*, each 2 L above), to afford 7 major fractions (Fr. 1–7) according to TLC profiles. Then, Fr. 1 (1.0 g) was successively subjected to RP-MPLC (YMC gel ODS-A-HG, MeOH/H_2_O, 20%–100%), Sephadex LH-20 CC (petroleum ether/CH_2_Cl_2_, 1:1), and preparative TLC to afford compounds **5** (34.1 mg), 6 (12.7 mg), and 7 (8.3 mg).

Compound **1** (63.3 mg) was obtained orderly by RP-MPLC (MeOH/H_2_O, 30%–80%), Sephadex LH-20 CC (MeOH) and purified by preparative TLC using CH_2_Cl_2_/EtOAc (20:1) from Fr. 2 (2.2 g). Fr. 7 (5.7 g) was subjected to RP-MPLC (MeOH/H_2_O, 20%–100%) and Sephadex LH-20 CC (petroleum ether/CH_2_Cl_2_/MeOH, 5:5:1) to gain compounds **2** (42.5 mg) and 3 (11.9 mg). Similarly, compound **4** (19.7 mg) was gained from Fr.4 (2.0 g) using the same method of compounds **2** and **3**.

### 3.4. Data for ***1**–**4***

*7βH-guaia-1(10)-en-12,8β-olide* (**1**): Colorless needles (acetone); ${\left[\alpha \right]}_{\text{D}}^{20}$ +74.9 (*c* 0.1, MeOH); mp 69 °C; IR ν_max_ 2961, 2933, 2874, 1774, 1734, 1706, 1616, 1456, 1380, 1331, 1240, 1216, 1185, 1001, 940, 730, 601 cm^−1^; ^1^H-NMR (methanol-*d*_4_, 400 MHz) and ^13^C-NMR (methanol-*d*_4_, 100 MHz) data, see Table 1 and Table 2; HRESIMS *m*/*z* 271.1323 [M + Na]^+^ (calcd for C_15_H_20_O_3_Na, 271.1310).

X-ray Crystallographic Analysis of compound **1**: The crystallographic data were collected with a Bruker APEX2 CCD and graphite monochromated Cu Kα radiation at Bruker SAINT. The structure was solved and refined by SHELXS-97, SHELXL-97, respectively. Compound **1** was crystallized from acetone to yield colorless needles. A single crystal of dimensions 0.18 × 0.11 × 0.06 mm^3^ was used for the X-ray measurements. Crystal data: C_15_H_20_O_3_, M = 248.31, space group C121, a = 17.6380 (8) Å, b = 6.6237 (4) Å, c = 12.0602 (7) Å, V = 1328.08 (13) Å^3^, Z = 4, Dcalcd = 1.242 mg/m^3^, R_1_ = 0.0478, wR_2_ =0.1265 [I > 2σ(I)], and the absolute structure parameter 0.1 (3). CCDC 1057220 contains the supplementary crystallographic data. Copy of the data can be obtained free of charge by applying to the Director at CCDC, 12 Union Road Cambridge CB2 1EZ, UK, Fax: +44-1223336033 or E-Mail: data_request@ccdc.cam.ac.uk.

*1,10-dioxo-4αH-5αH-7βH-11αH-1,10-secoguaia-2(3)-en-12,8β-olide* (**2**): Colorless oil; ${\left[\alpha \right]}_{\text{D}}^{20}$ +56.6 (*c* 0.1, MeOH); IR ν_max_ 2926, 1771, 1701, 1588, 1457, 1381, 1361, 1173, 1042, 975, 812 cm^−1^; ^1^H-NMR (CDCl_3_, 600 MHz) and ^13^C-NMR (CDCl_3_, 150 MHz) data, see Table 1 and Table 2; HRESIMS *m*/*z* 287.1251 [M + Na]^+^ (calcd for C_15_H_20_O_3_Na, 287.1259).

*1β-Hydroxy-4βH-5βH-7βH-11αH-8,9-secoguaia-9(10)-en-8,12-olide* (**3**): Colorless oil; ${\left[\alpha \right]}_{\text{D}}^{20}$ +51.0 (*c* 0.1, MeOH); IR ν_max_ 3447, 2927, 2873, 1757, 1669, 1457, 1380, 1175, 1021, 899 cm^−1^; ^1^H-NMR (CDCl_3_, 600 MHz) and ^13^C-NMR (CDCl_3_, 150 MHz) data, see Table 1 and Table 2; HRESIMS *m*/*z* 291.1594 [M + Na]^+^ (calcd for C_15_H_20_O_3_Na, 291.1572).

*1α-Hydroxy-4α,10α-dimethyl-5βH-octahydro-azulen-8-one* (**4**): Colorless oil; ${\left[\alpha \right]}_{\text{D}}^{20}$ +96.0 (*c* 0.1, MeOH); IR ν_max_ 3447, 2954, 2871, 1698, 1457, 1265, 1111, 1013, 978, 896 cm^−1^; ^1^H-NMR (CDCl_3_, 400 MHz) and ^13^C-NMR (CDCl_3_, 100 MHz) data, see Table 1 and Table 2; HRESIMS *m*/*z* 219.1387 [M + Na]^+^ (calcd for C_15_H_20_O_3_Na, 219.1361).

### 3.5. Anti-Inflammatory Assay

RAW 264.7 cells were maintained in Dulbecco’s Modified Eagles Medium containing 10% fetal bovine serum in a humid atmosphere of 5% CO_2_ at 37 °C and plated into 96-well plates at a density of approximately 1 × 10^5^ cells per well. The cells were pretreated with different concentrations of the detected compounds for 2 h and then incubated for 12 h with or without LPS (2 μg/mL). The nitrite content in the culture supernatant was measured by the Griess reaction. The IC_50_ value was calculated by GraphPad Prism software with the inhibition rates of different concentrations. Moreover, the formula of inhibition rate is (OD_Model_ − OD_Compound_)/(OD_Model_ − OD_Control_) × 100%. Cell viability was measured by Cell Counting Kit-8 (CCK-8, Dojindo, Kumamoto, Japan) method.

## 4. Conclusions

A systematic chemical search was performed and resulted in the separations of three new sesquiterpenoids and one sesquiterpenoid derivative, together with three known sesquiterpenoids from Chinese eaglewood. The structures of the new compounds were identified based on detailed spectroscopic analysis and published analogues. In addition, the stereo configuration of compound **1** was confirmed by Cu Kα X-ray crystallographic experiment. Besides, the new compound **2** showed significant inhibitory activity against LPS induced NO production in RAW 264.7 cells.

## Figures and Tables

**Table 1:** ^1^H-NMR spectroscopic data of compounds **1**–**4** (*J* in Hz) ^a^.

Position	1	2	3	4
1				
2a		6.09 dd (6.0,2.4)	1.67 m	1.53 m
2b			2.01 m	1.97 m
3a	2.44 m	7.55 dd (6.0,2.4)	1.56 m	1.29 m
3b	2.04 dd (16.0,5.6)		2.00 m	1.96 m
4	2.36 m	2.6 m	2.38 m	2.77 m
5	2.94 m	1.91 m	2.62 m	1.66 m
6a	1.95 m	1.90 m	1.65 m	1.27 m
6b	1.31 m	1.66 m		1.72 m
7a	1.90 m	2.25 m	2.46 m	2.41 m
7b				2.54 m
8	3.86 td (9.6,3.6)	4.53 m		
9a	2.73 m	2.89 m	4.90 s	2.19 dd (15.2,1.2)
9b			5.07 s	2.71 m
10				2.09 m
11	2.45 m	2.37 m	2.41 m	0.98 d (7.2)
12a			4.12 t (8.4)	1.08 d (6.8)
12b			4.39 t (8.4)	
13a	1.21 d (6.8)	1.30 d (7.2)	3.74 dd (10.8,4.2)	
13b			3.81 dd (10.8,4.2)	
14	0.96 d (6.8)	1.25 d (7.2)	1.06 d (7.2)	
15	2.31 d (2.4)	2.22 brs	1.81 s	
				1-OH 4.13 d (0.4) ^b^

^a^ Data were measured at 600 MHz for compound **2** (in CDCl_3_), compound **3** (in CDCl_3_) and at 400 MHz for compound **1** (in methanol-d_4_), compound **4** (in CDCl_3_); ^b^ Data were measured in DMSO at 400 MHz for compound **4**.

**Table 2:** ^13^C-NMR spectroscopic data for compounds **1**–**4** ^a^.

Position	1	2	3	4
1	138.5 s	210.8 s	85.8 s	86.9 s
2	209.4 s	132.2 d	39.0 t	29.7 t
3	49.0 t	168.2 d	32.1 t	28.9 t
4	32.5 d	43.8 d	33.4 d	35.6 d
5	47.2 d	50.4 d	44.0 d	57.0 d
6	30.2 t	33.5 t	25.0 t	19.7 t
7	56.0 d	45.6 d	38.8 d	41.5 t
8	81.4 d	78.8 d	179.6 s	213.9 s
9	44.0 t	47.8 t	110.1 t	46.2 t
10	150.3 s	205.2 s	149.6 s	38.5 d
11	43.5 d	41.9 d	44.8 d	15.8 q
12	180.7 s	178.1 s	68.6 t	18.5 q
13	12.7 q	15.2 q	61.7 t	
14	16.8 q	19.3 q	17.8 q	
15	22.9 q	30.9 q	19.5 q	

^a^ Data were measured at 150 MHz for compound **2** (in CDCl_3_), compound **3** (in CDCl_3_) and at 400 MHz for compound **1** (in methanol-*d*_4_), compound **4** (in CDCl_3_).

## Supplementary Materials

Supplementary materials can be accessed at: http://www.mdpi.com/1420-3049/21/3/281/s1.

## Abbreviations

The following abbreviations are used in this manuscript:

IR: Infrared
NMR: Nuclear magnetic resonance
HRESIMS: High resolution electrospray ionization mass spectroscopy
LPS: Lipopolysaccharide
UPLC: Ultra-performance liquid chromatography
EtOAc: Ethyl acetate
MeOH: Methanol
CC: Column chromatography
