# Supplementary material for: Three New Sesquiterpenoids and One New Sesquiterpenoid Derivative from Chinese Eaglewood
Source: Molecules. 2016 Feb 27;21(3):281. doi: 10.3390/molecules21030281 (PMC6274321; doi:10.3390/molecules21030281)
Supplement: Supplementary file 1 [file molecules-21-00281-s001.pdf]

Supplementary Materials: Three New Sesquiterpenoids and One New Sesquiterpoid Derivative from Chinese Eaglewood

Huan Zhao, Qinghua Peng, Zhuzhen Han, Li Yang andZhengtao Wang

Table S1. Anti-inflammatory activity of the new compounds.

Compound	IC_{50} (μM)
**1**	>100
**2**	8.1
**3**	>100
**4**	>100
Aminoguanidine hydrochloride [a]	11.6

[a] positive control.

Table S2. Crystallographic data of Compound (**1**).

Indentification Code	cu_dm15185_0m
Empirical formula	$C_{15}H_{20}O_3$
Formula weight	248.31
Temperature	296.15 K
Wavelength	1.54178 Å
Crystal system	Monoclinic
Space group	C 1 2 1
Unit cell dimensions	a = 17.6380(8) Å, α = 90°; b = 6.6237(4) Å,<br>β = 109.510(4)°; c = 12.0602(7) Å, γ = 90°
Volume	1328.08 (13) $Å^3$
Z	4
Calculated density	1.242 mg/m^3
Absorption coefficient	0.685 mm^{-1}
F(000)	536
Crystal size	0.18 × 0.11 × 0.06 mm^3
Theta range for data collection	5.321 to 69.651°
Limiting indices	−19≤ h ≤21<br>−7≤ k ≤ 5<br>−14 ≤ l ≤ 14
Reflections collected/unique	4997/1923[R(int) = 0.0514]
Completeness to theta = 67.679°	99.2%
Absorption correction	Semi-empirical from equivalents
Max. and min. transmission	0.7533 and 0.4608
Refinement method	Full-matrix least-squares on F^2
Data/restraints/parameters	1923/1/166
Goodness-of-fit on F^2	1.029
Final R indices [I>2σ(I)]	R_1 = 0.0478, wR_2 = 0.1265
R indices (all data)	R_1 = 0.0504, wR_2 = 0.1315
Absolute structure parameter	0.1(3)
Largest diff. peak and hole	0.121 and −0.162 e·$Å^{-3}$

Crystallographic data for have been deposited at the Cambridge Crystallographic Data Centre (deposition No. CCDC 928251). Copies of these data can be obtained free of charge via www.ccdc.cam.ac.uk/conts/retrieving.html or from the Cambridge Crystallographic Data Centre, 12, Union Road, Cambridge CB21EZ, UK. [Fax: +44-1223-336-033; or email: deposit@ccdc.cam.ac.uk].

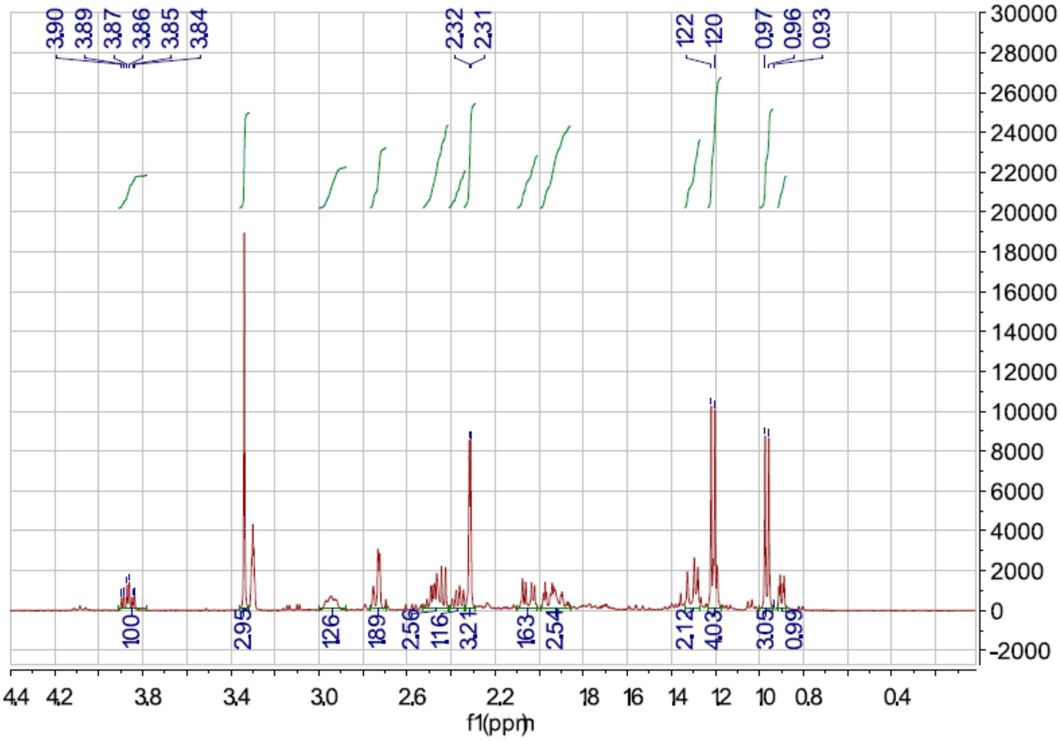

Figure S1. ^{1}H NMR spectrum of compound (**1**) (CD_3OD, 400 MHz).

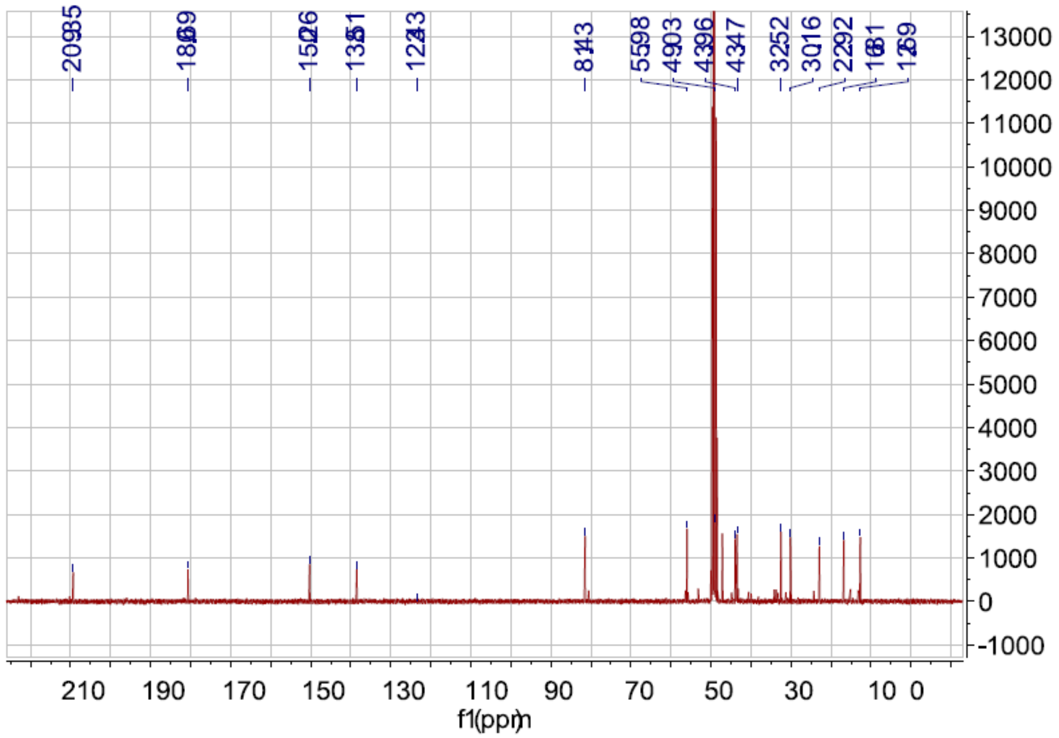

Figure S2. ^{13}C NMR spectrum of compound (**1**) (CD_3OD, 100 MHz).

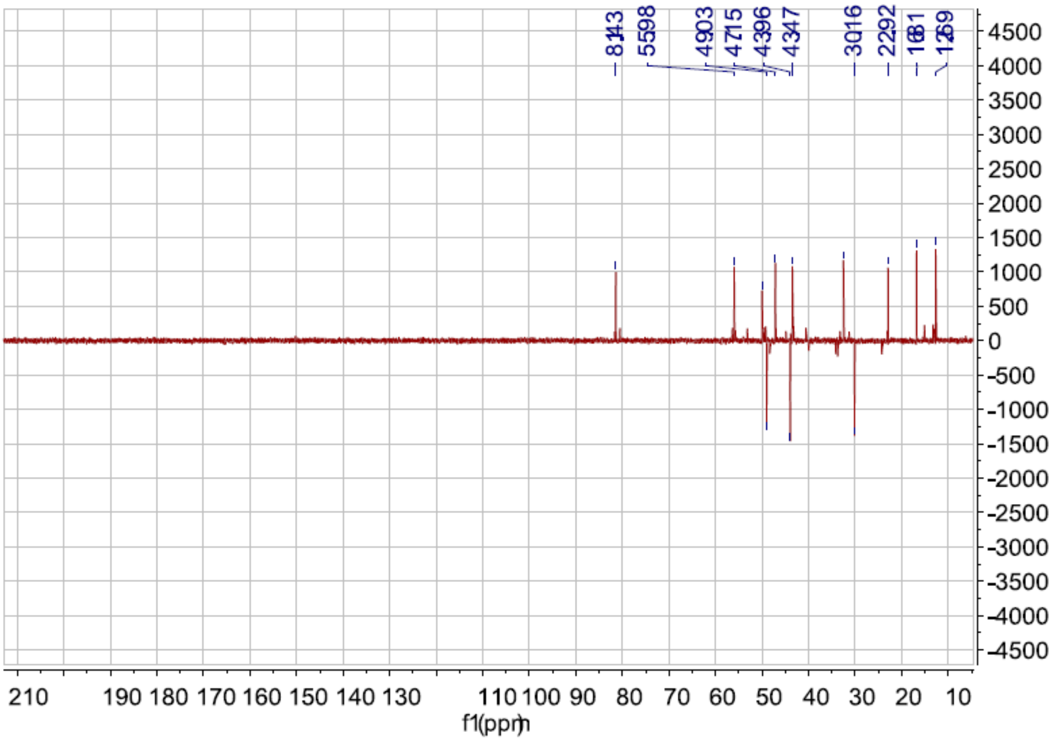

Figure S3. DEPT spectrum of compound (**1**).

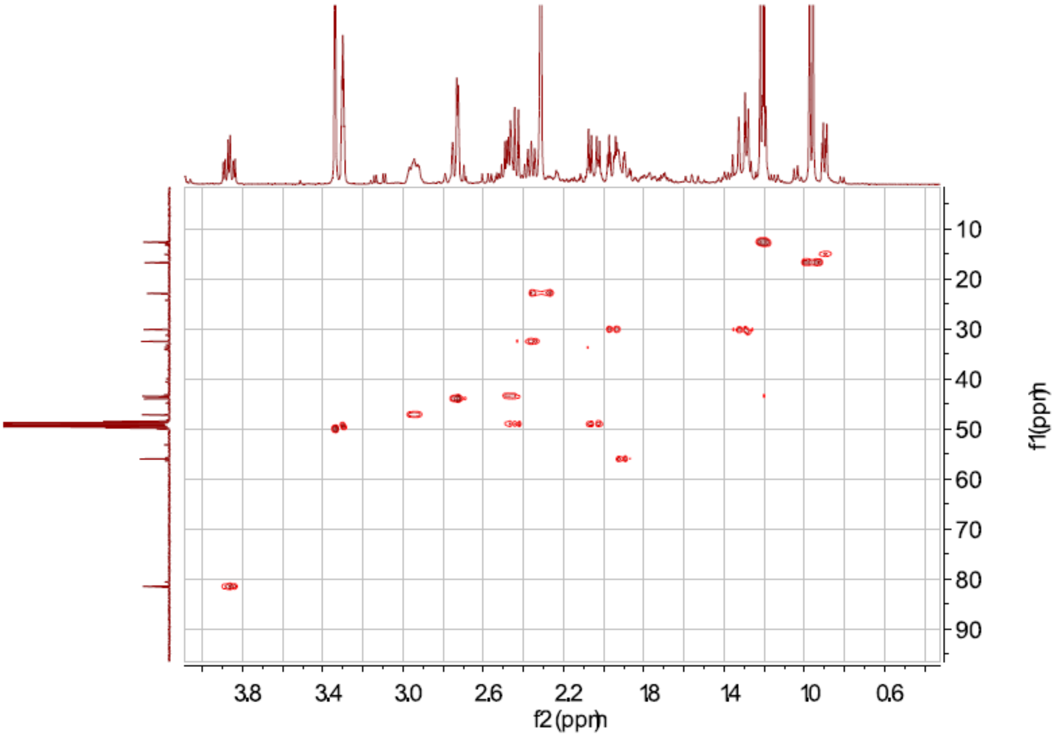

Figure S4. HSQC spectrum of compound (**1**).

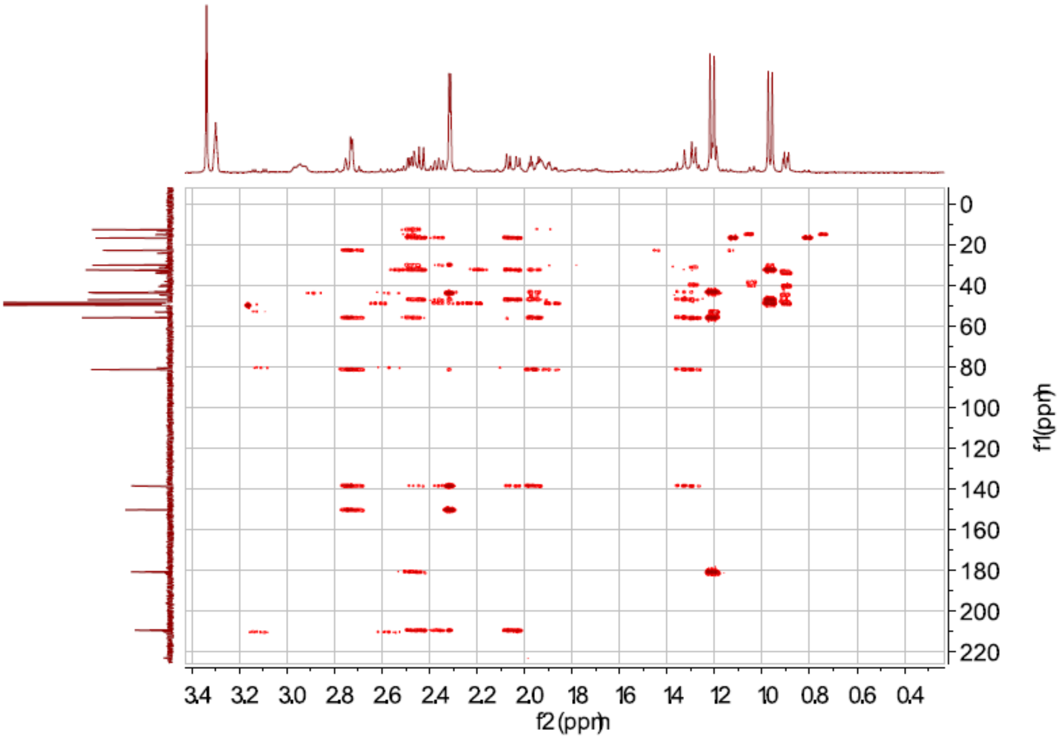

Figure S5. HMBC spectrum of compound (**1**).

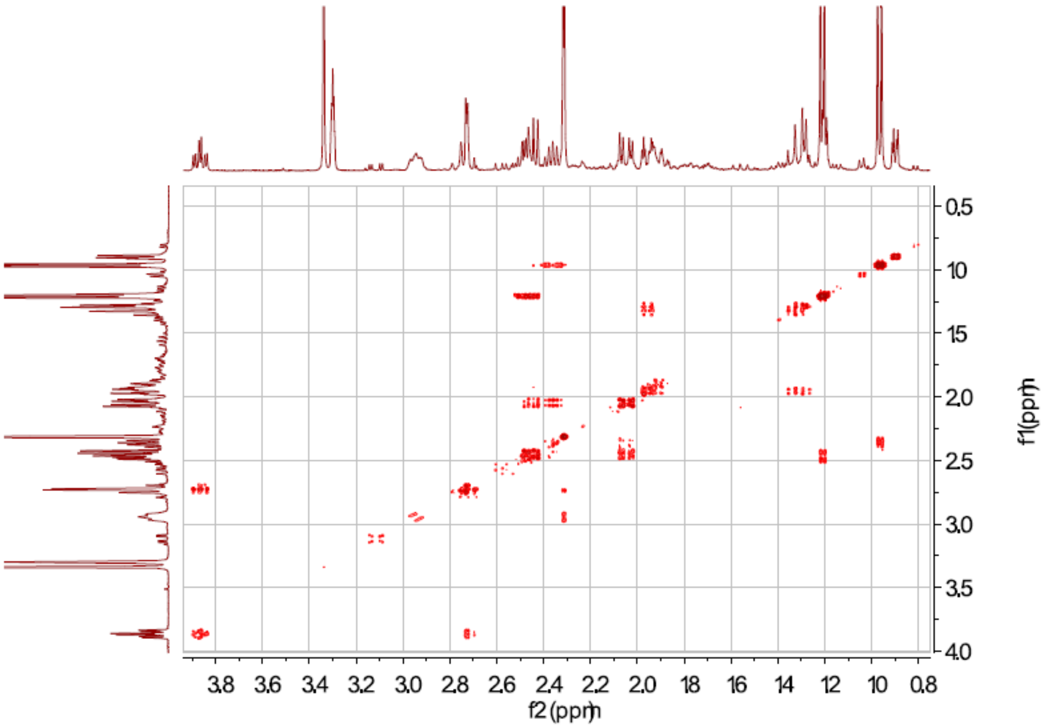

Figure S6.^{1}H-^{1}H COSY spectrum of compound (**1**).

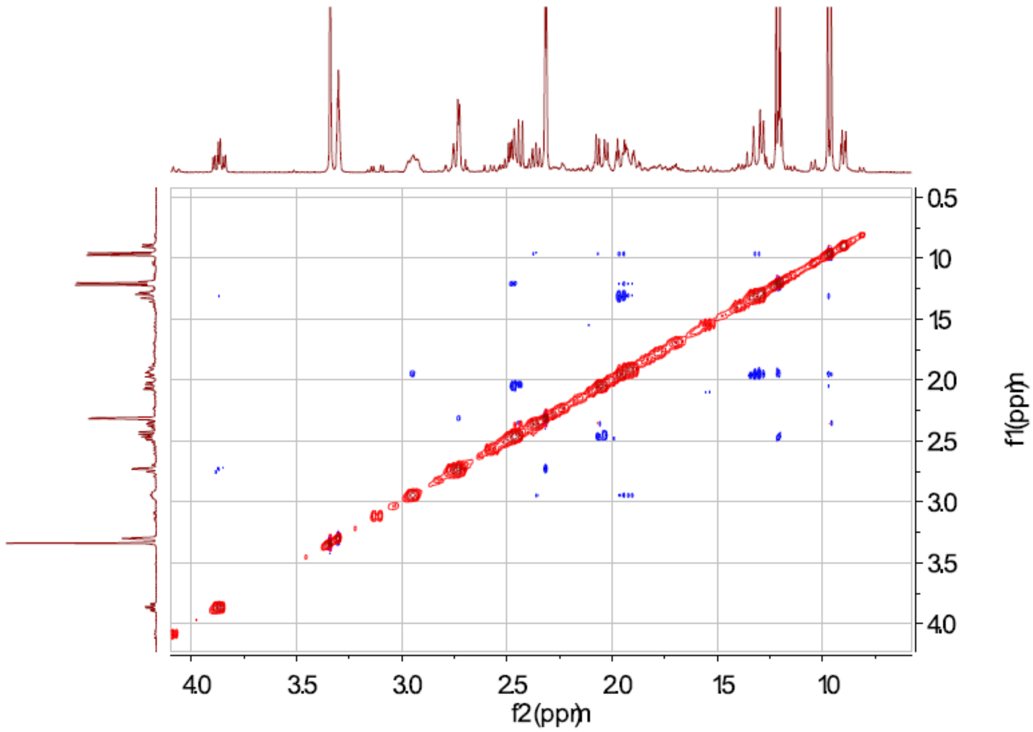

Figure S7. NOESY spectrum of compound (**1**).

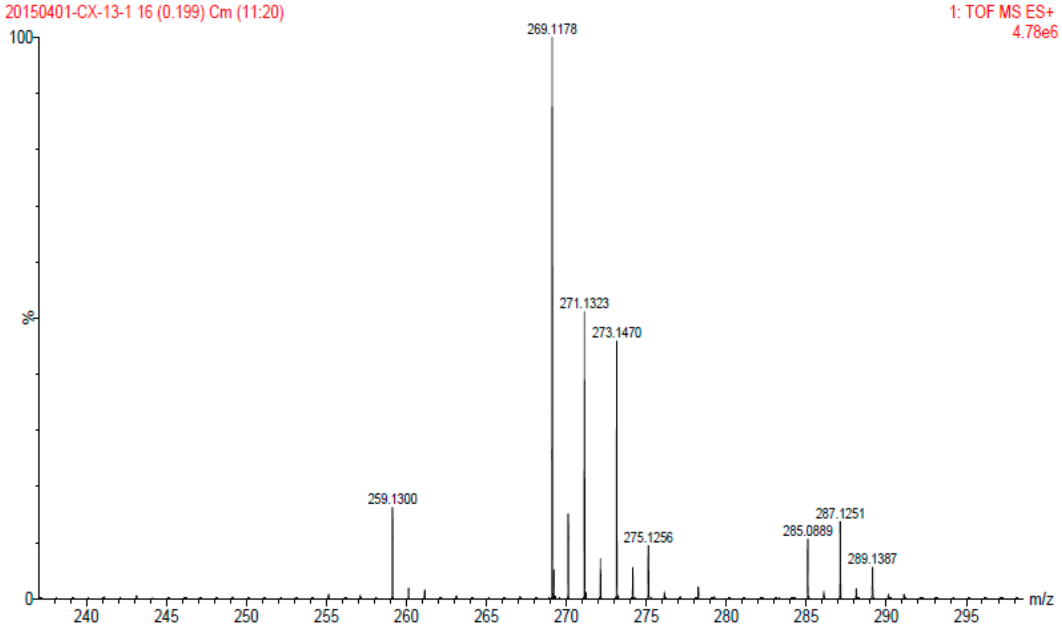

Figure S8. HR-ESIMS spectrum of compound (**1**).

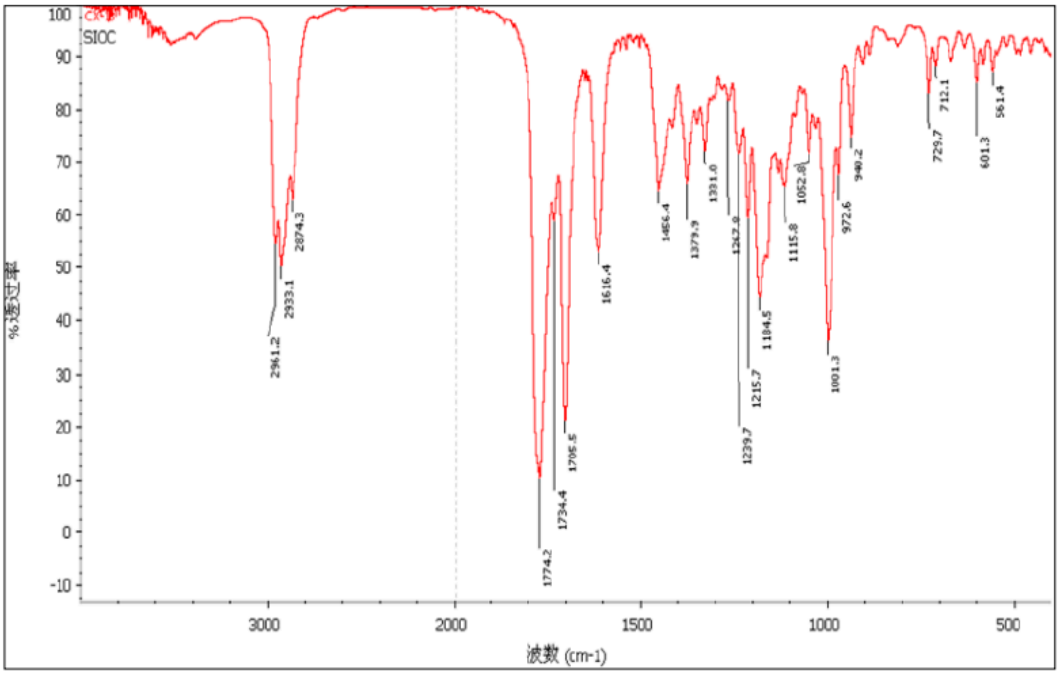

Figure S9. IR of compound (**1**).

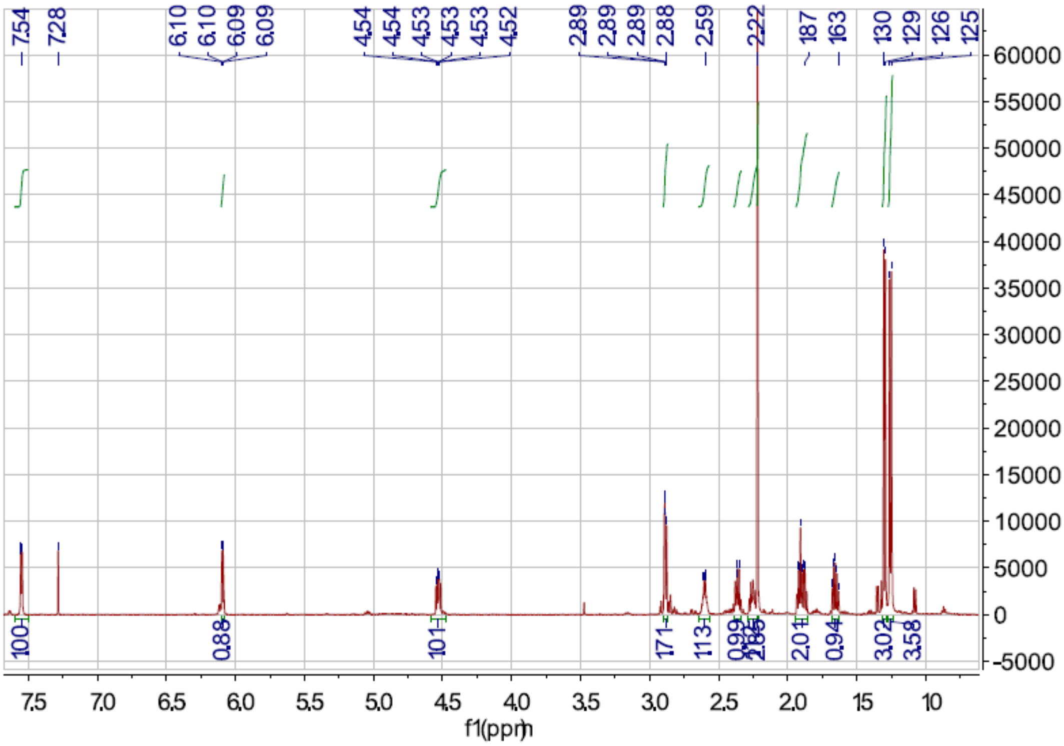

Figure S10.^{1}H NMR spectrum of compound (**2**) ($CDCl_3$, 600 MHz).

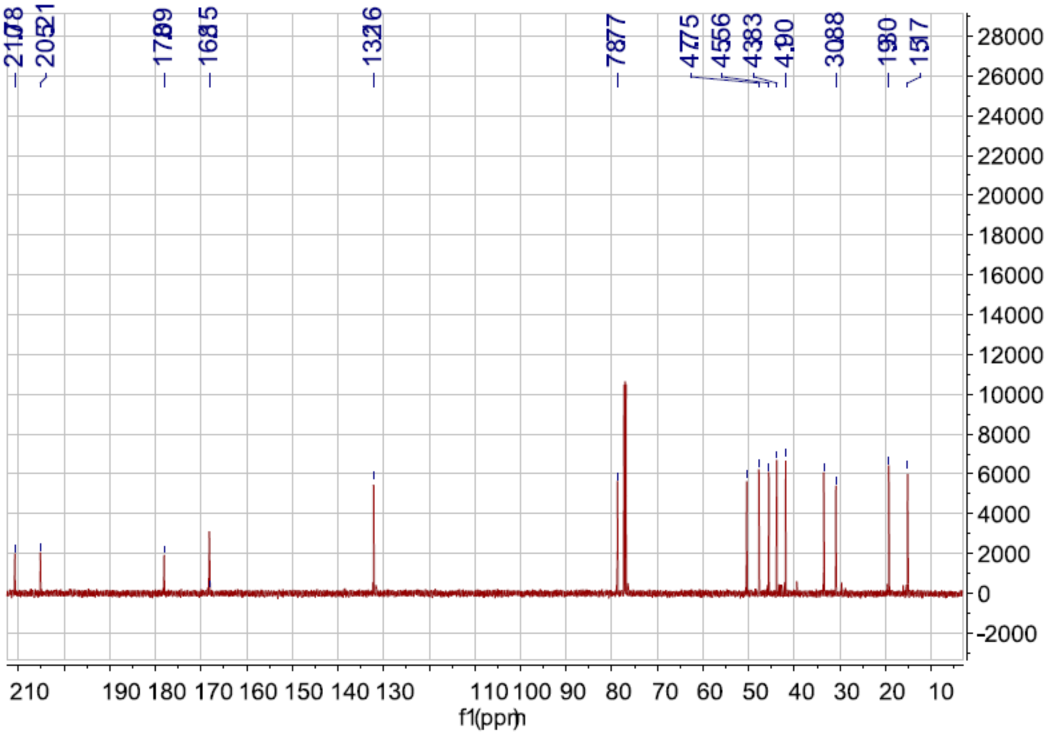

Figure S11. ^{13}C NMR spectrum of compound (**2**) ($CDCl_3$, 150 MHz).

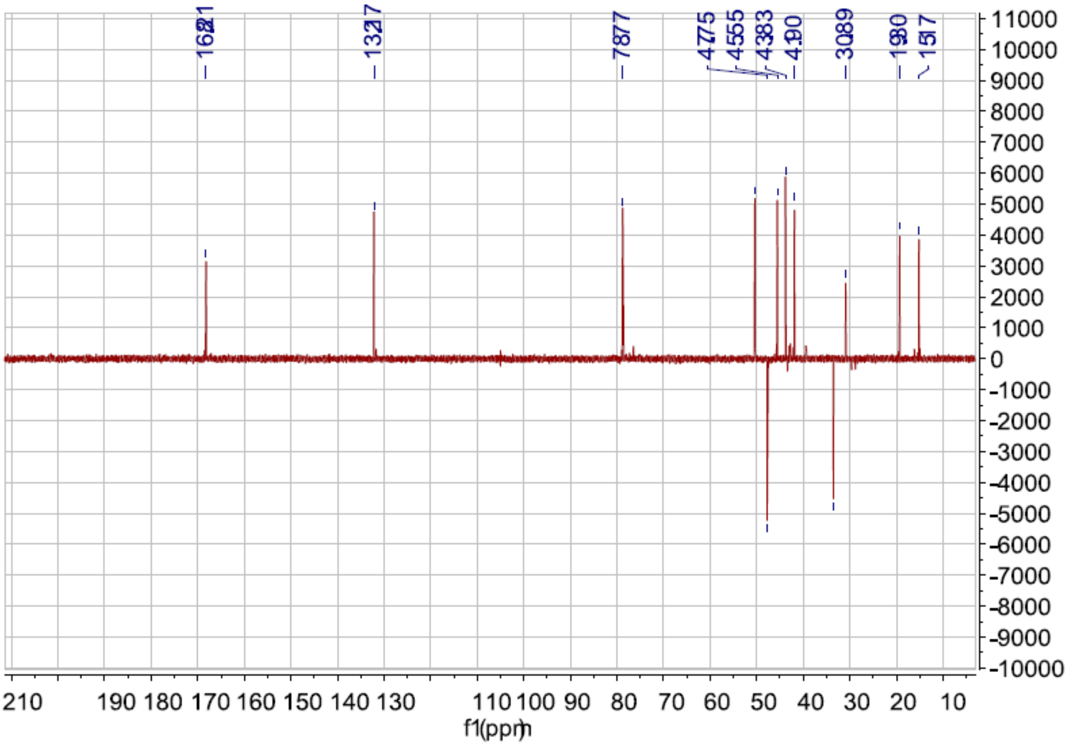

Figure S12. DEPT spectrum of compound (**2**).

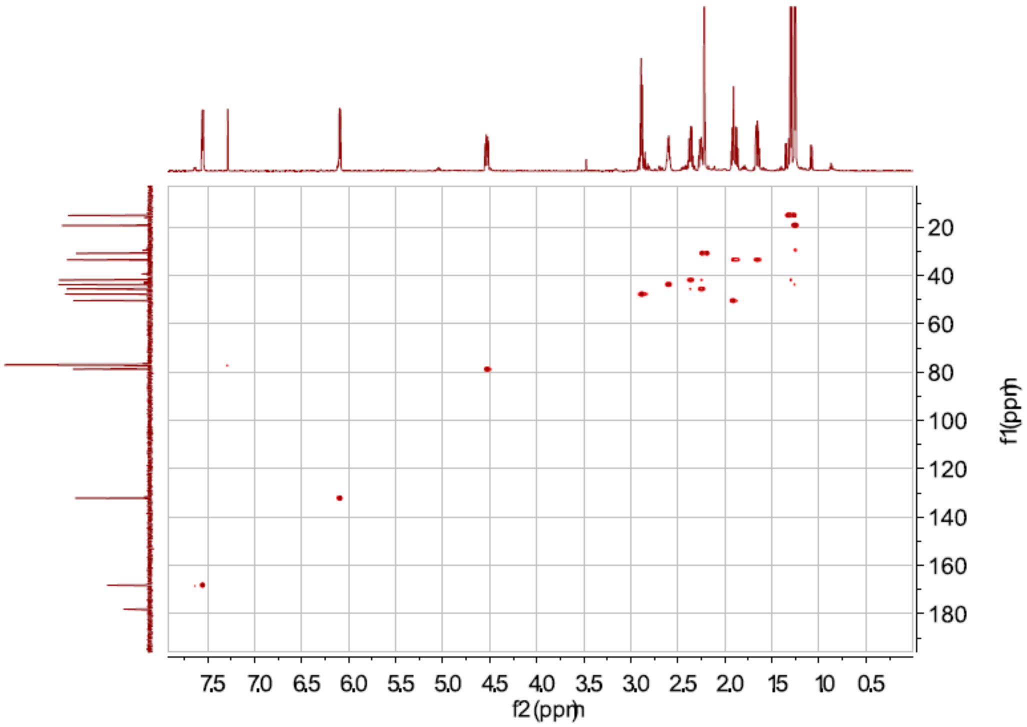

Figure S13. HSQC spectrum of compound (**2**).

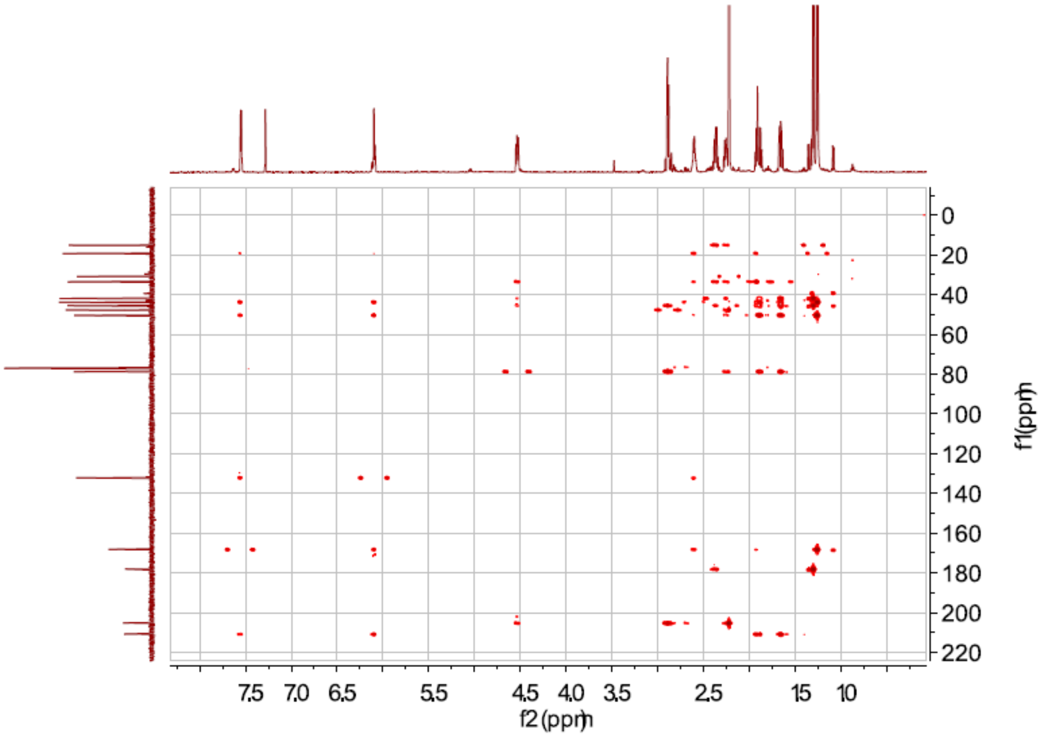

Figure S14. HMBC spectrum of compound (**2**).

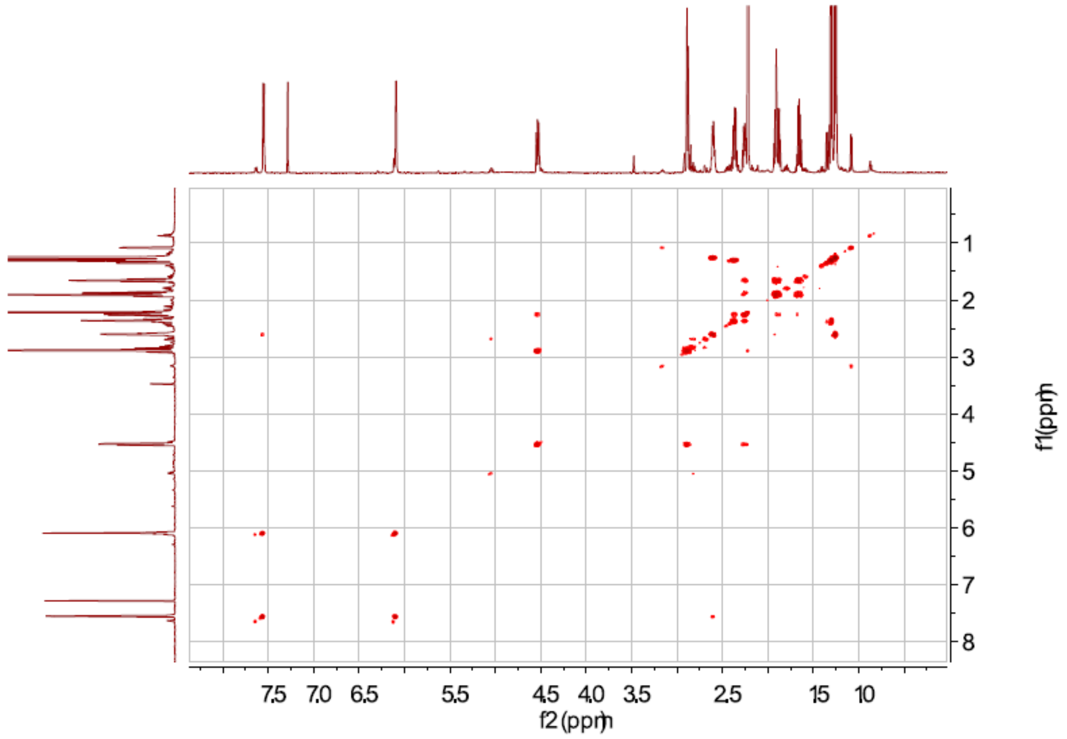

Figure S15. ^1H-^1H COSY spectrum of compound (**2**).

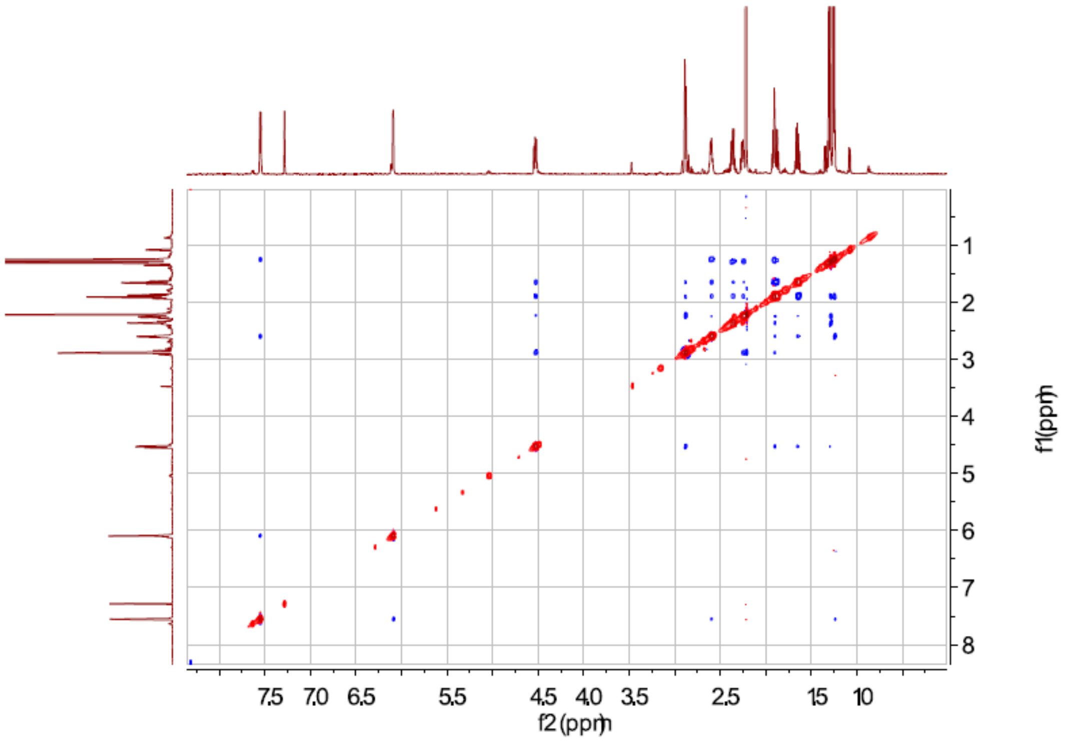

Figure S16. NOESY spectrum of compound (**2**).

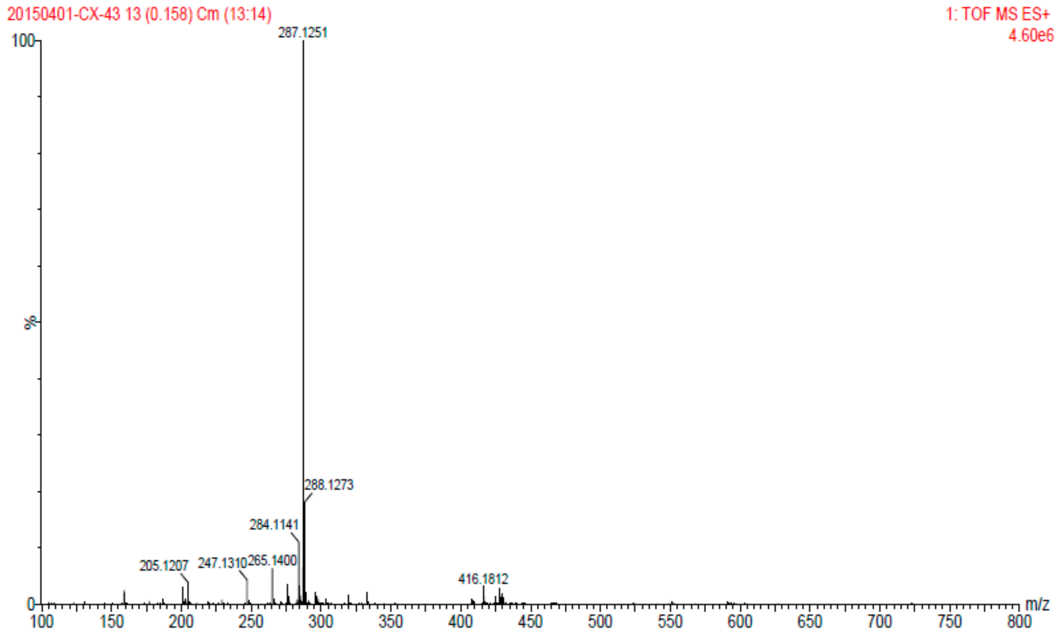

Figure S17. HR-ESIMS spectrum of compound (**2**).

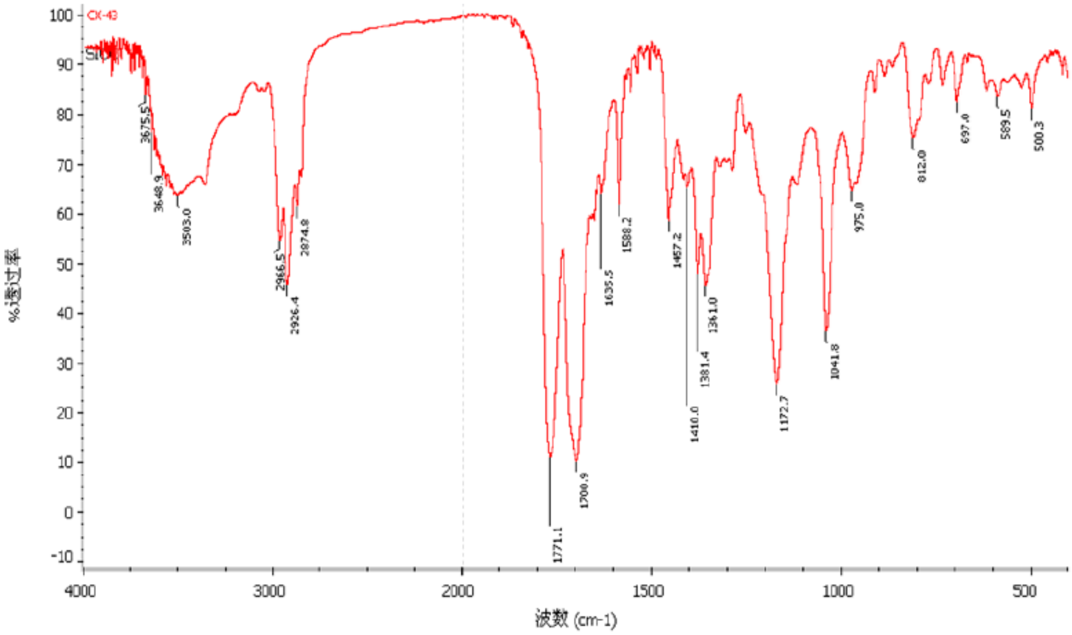

Figure S18. IR of compound (**2**).

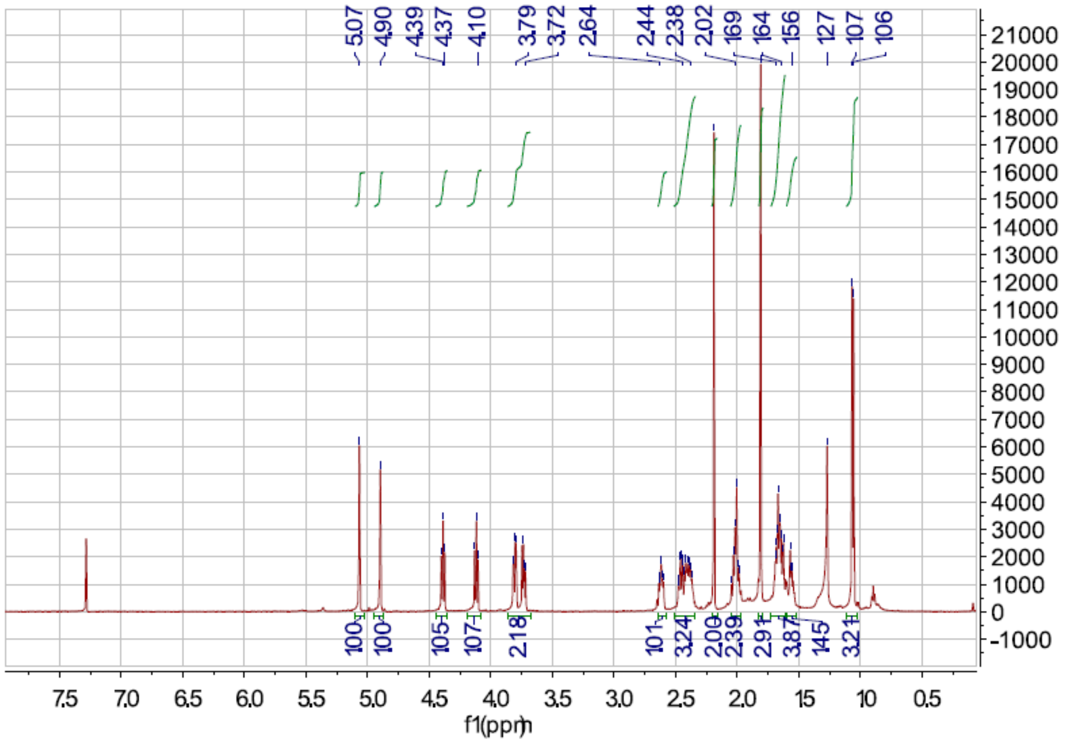

Figure S19.^{1}H NMR spectrum of compound (**3**) ($CDCl_3$, 600 MHz).

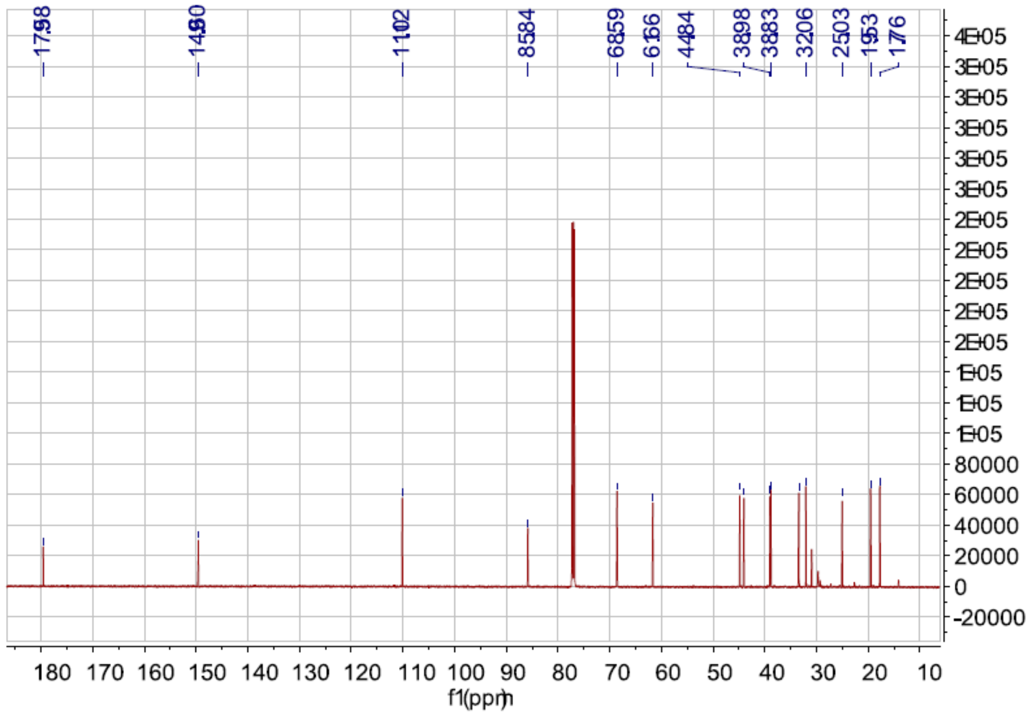

Figure S20.^{13}C NMR spectrum of compound (**3**) ($CDCl_3$, 150 MHz).

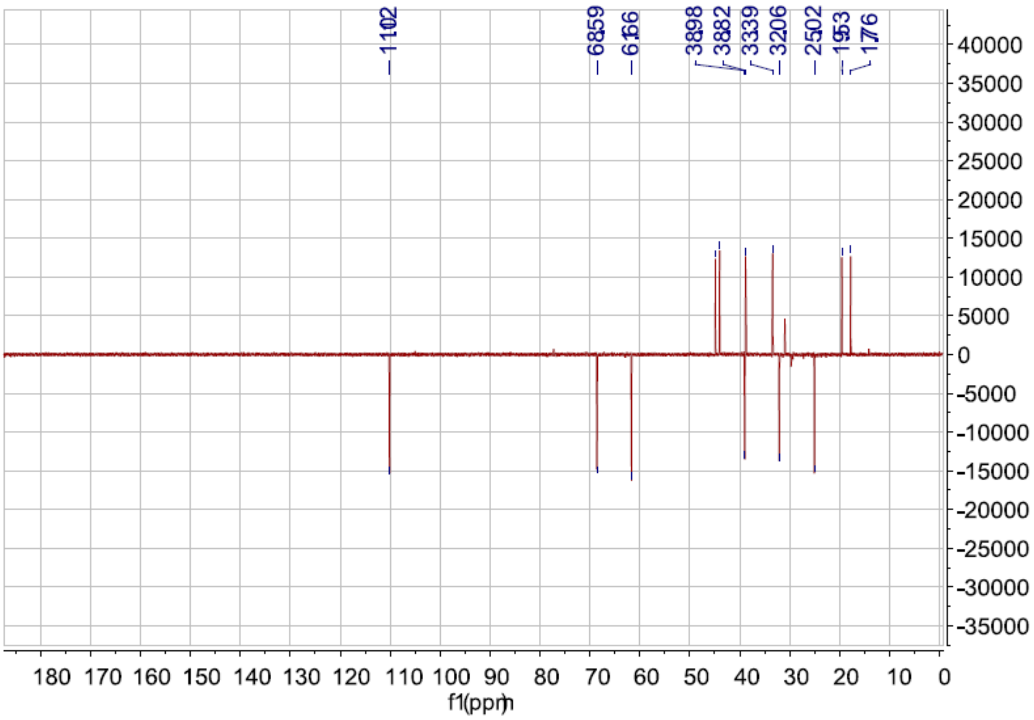

Figure S21. DEPT spectrum of compound (**3**).

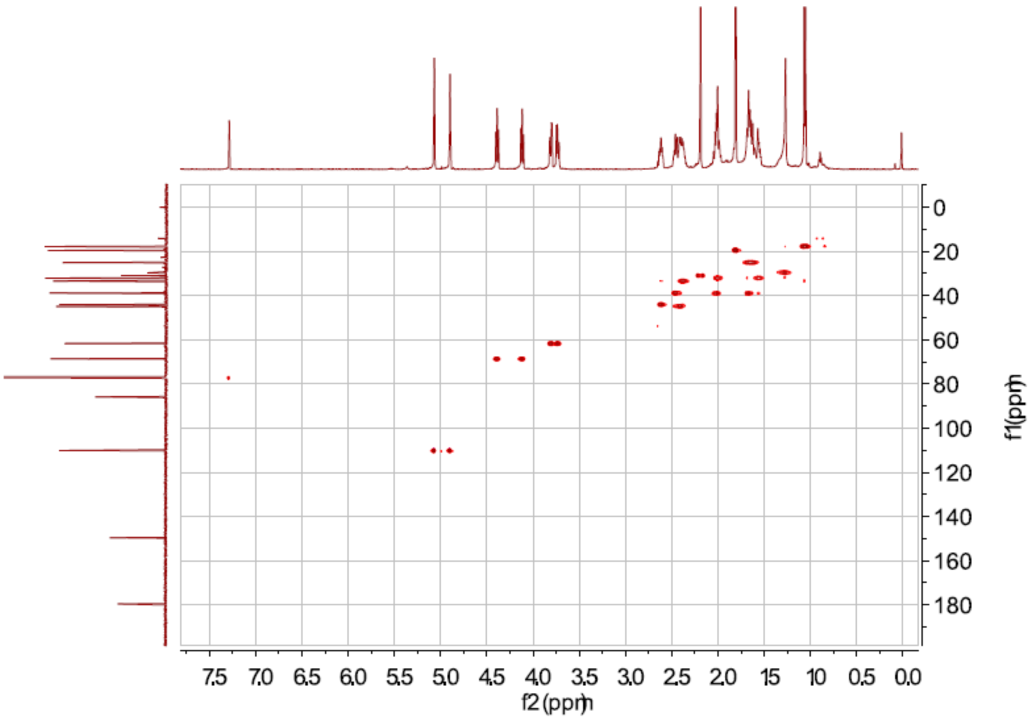

Figure S22. HSQC spectrum of compound (**3**).

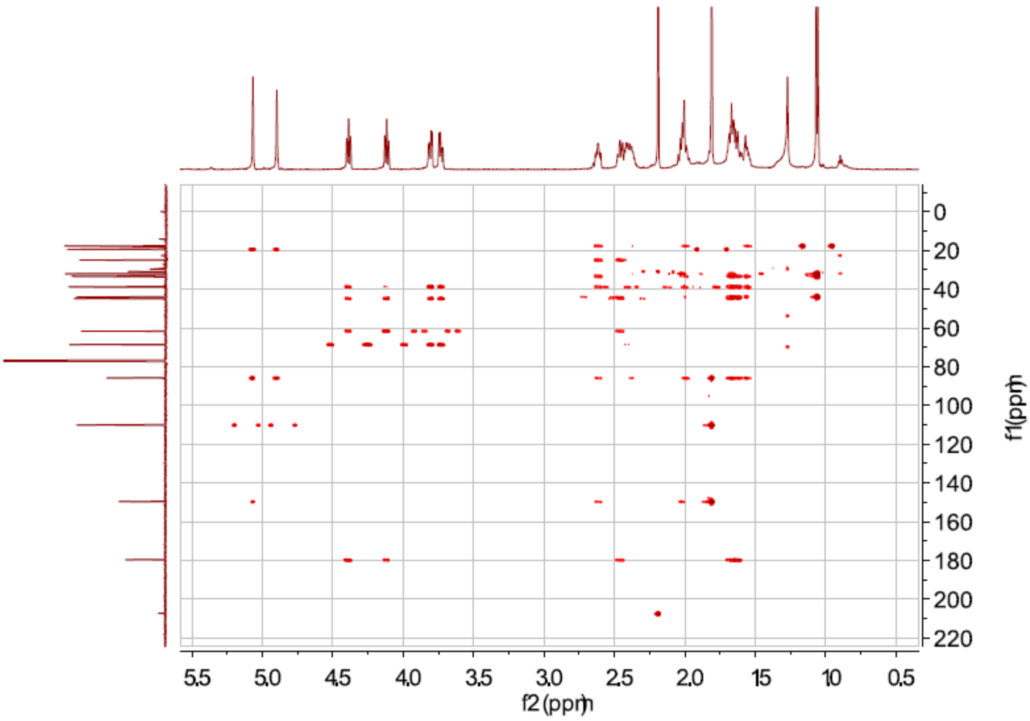

Figure S23. HMBC spectrum of compound (**3**).

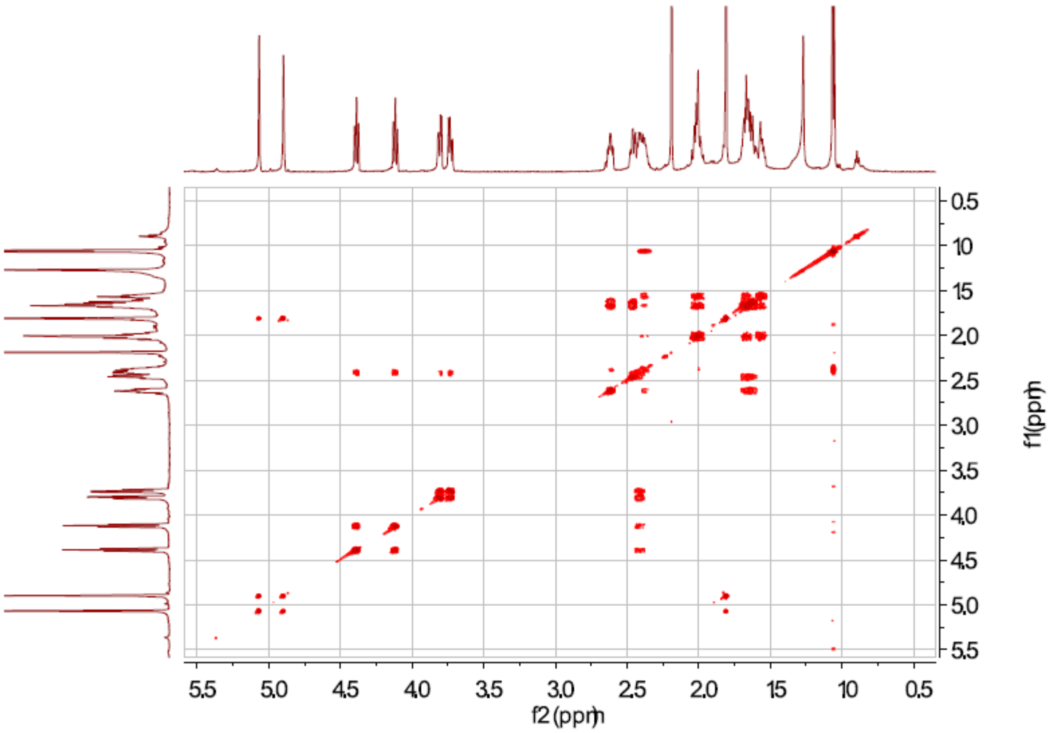

Figure S24. ^{1}H-^{1}H COSY spectrum of compound (**3**).

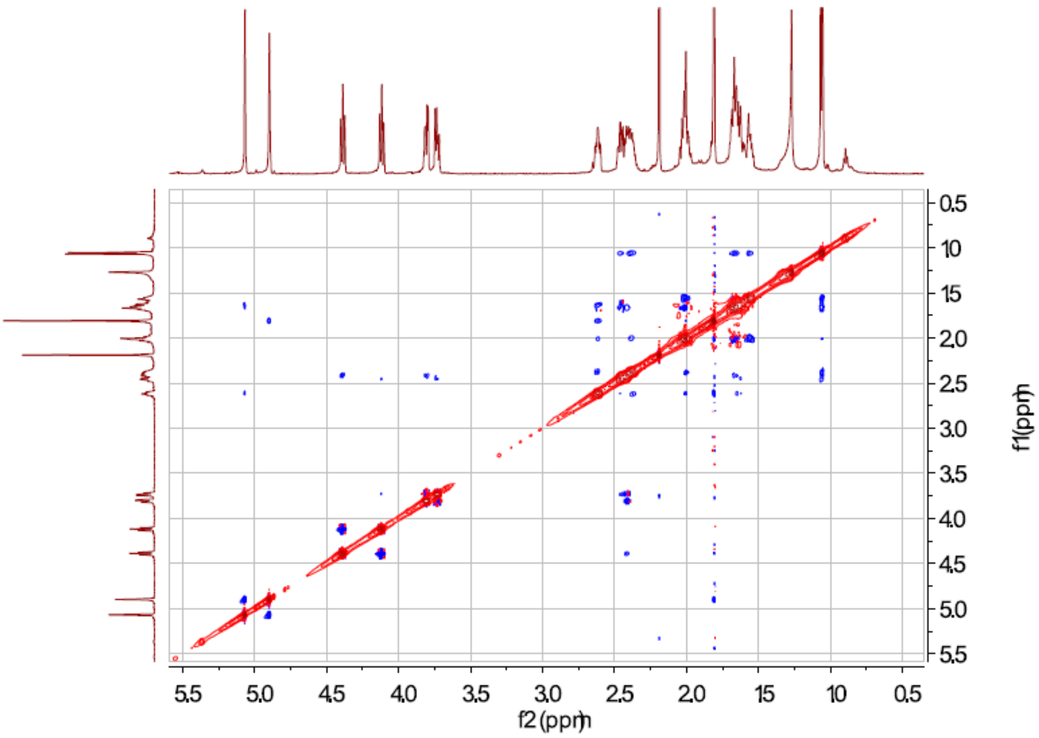

Figure S25. NOESY spectrum of compound (**3**).

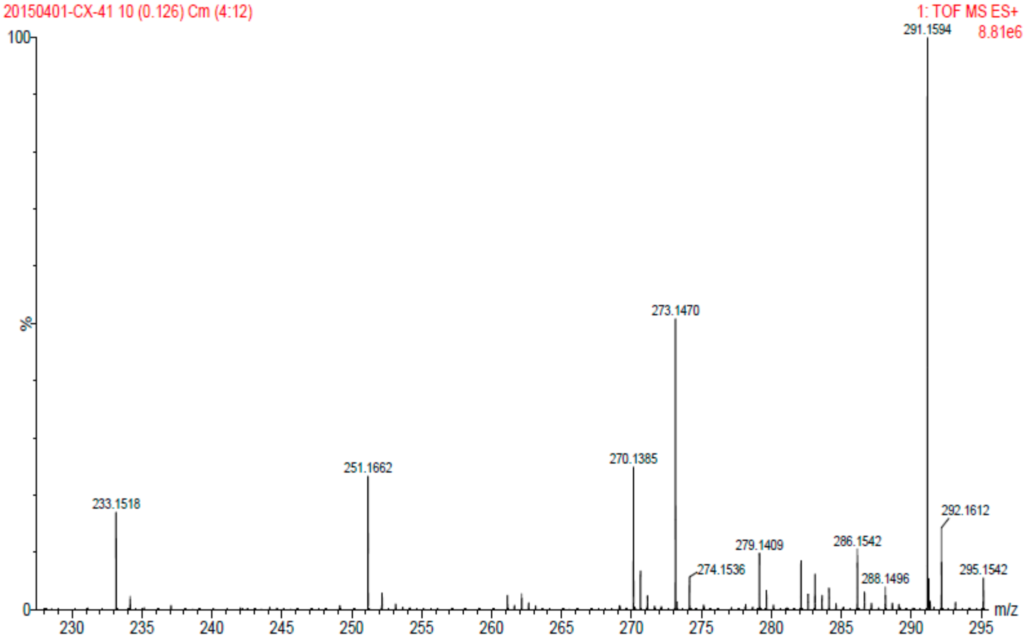

Figure S26. HR-ESIMS spectrum of compound (**3**).

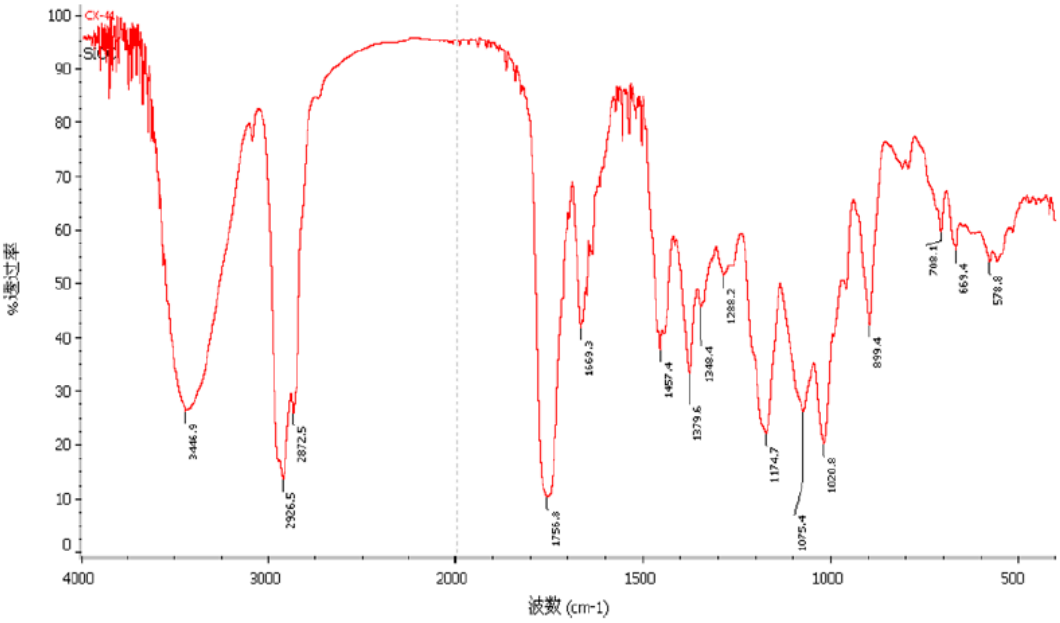

Figure S27. IR of compound (**3**).

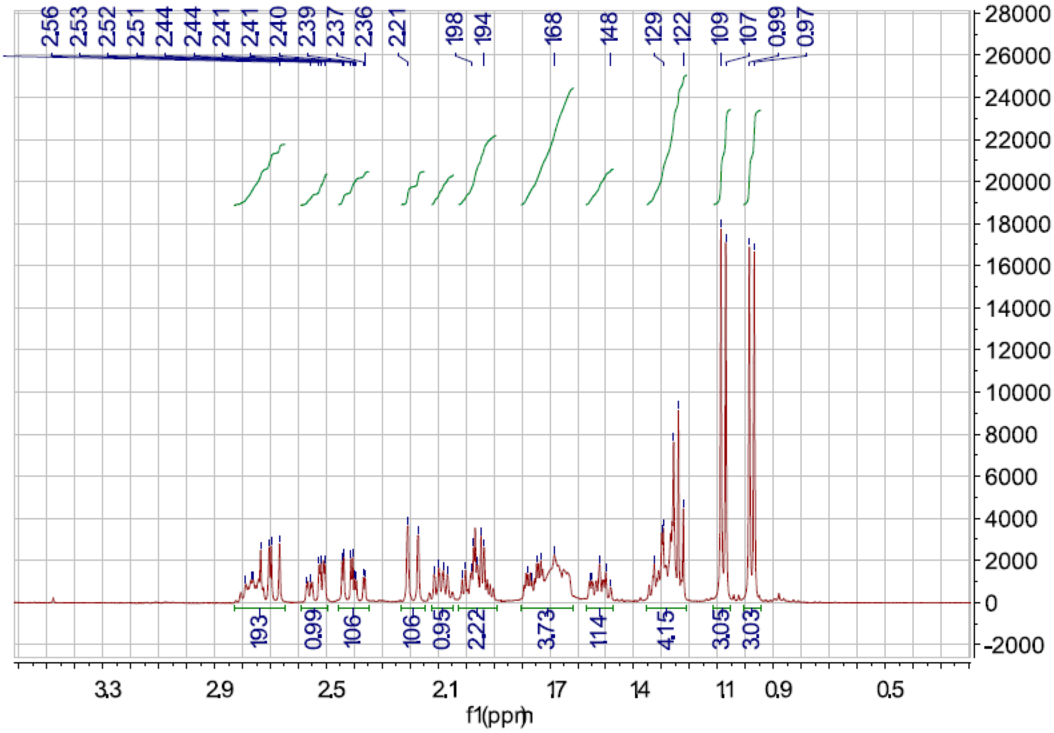

Figure S28. ^{1}H NMR spectrum of compound (**4**) ($CDCl_3$, 400 MHz).

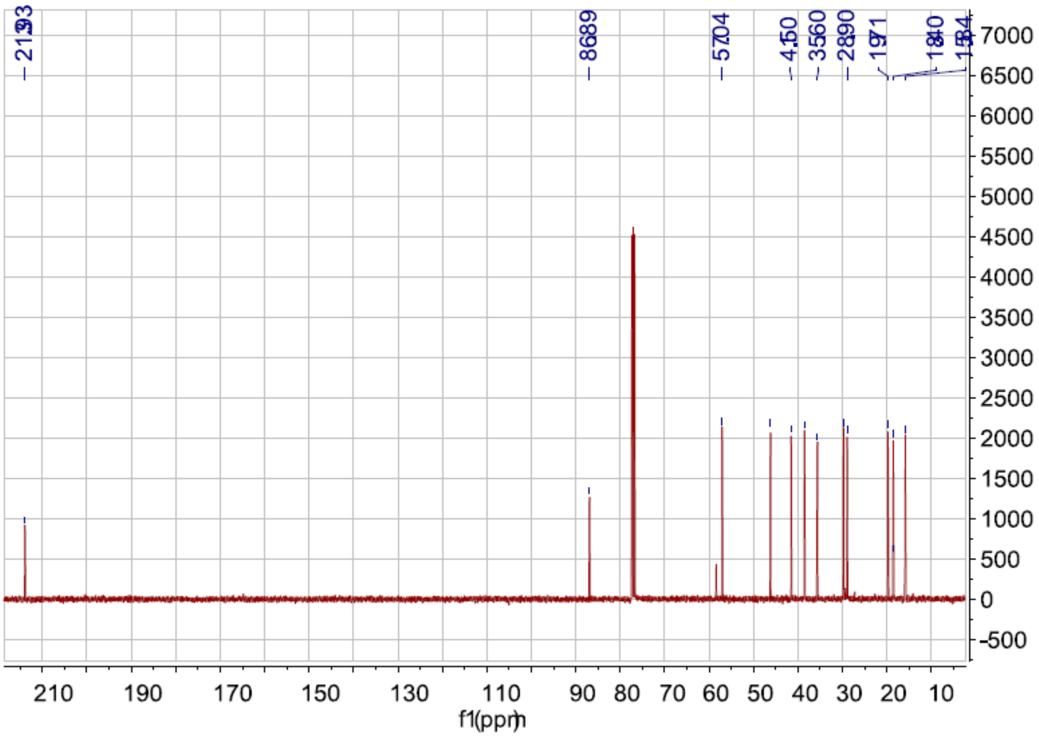

Figure S29. ^{13}C NMR spectrum of compound (**4**) ($CDCl_3$, 100 MHz).

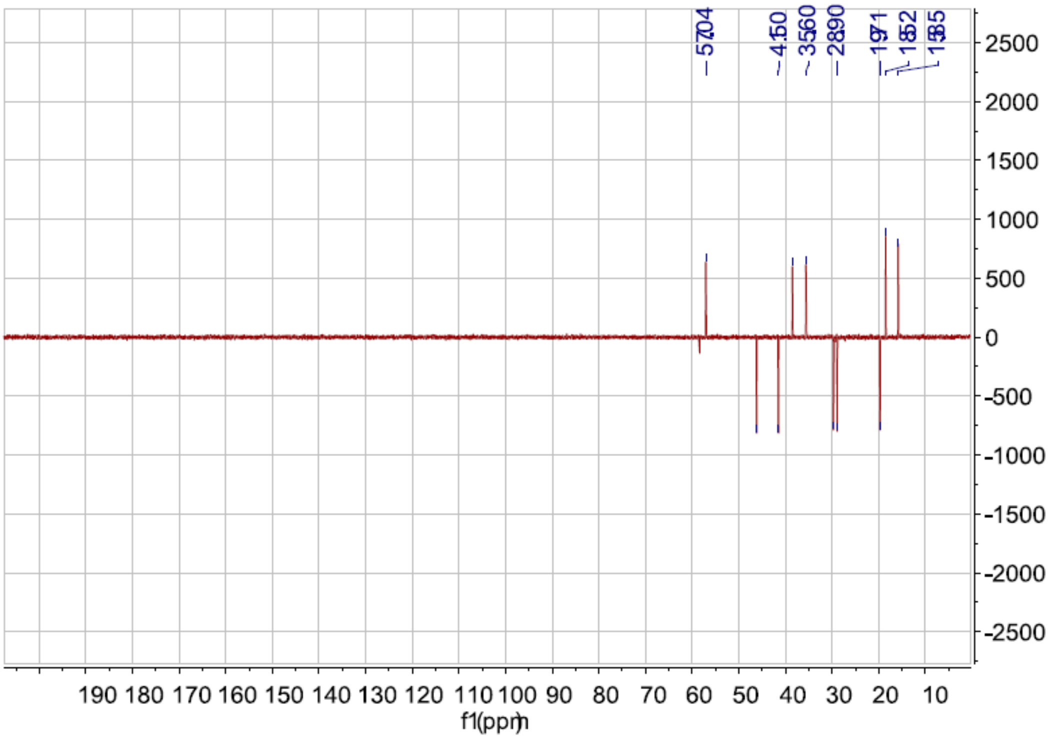

Figure S30. DEPT spectrum of compound (**4**).

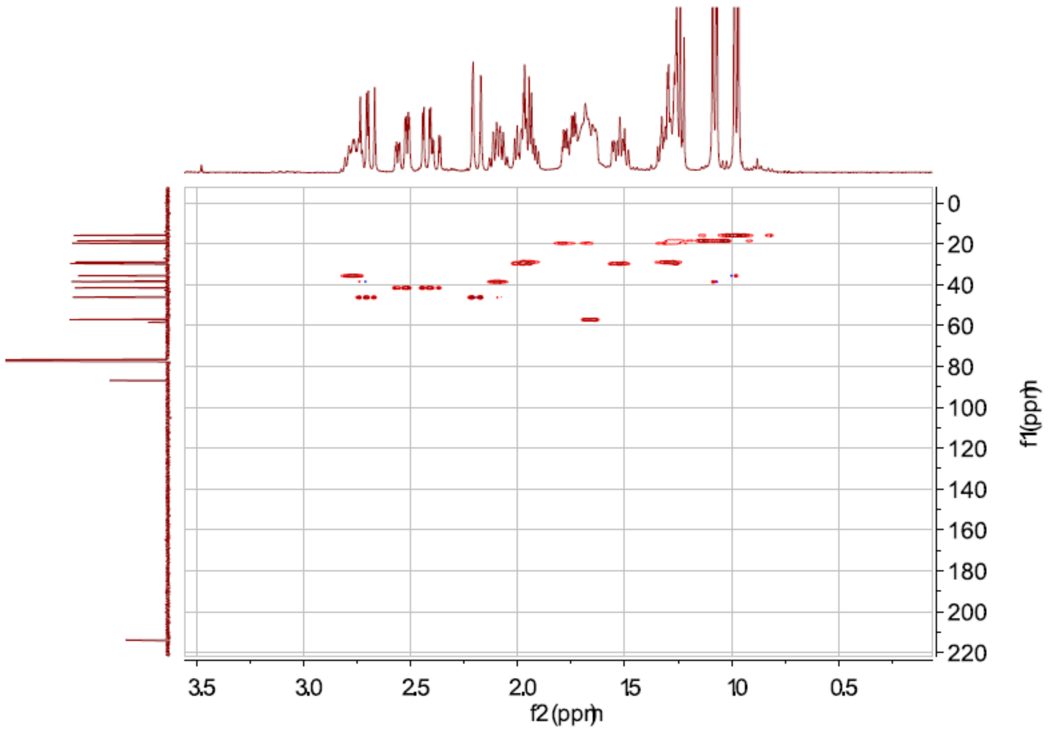

Figure S31. HSQC spectrum of compound (**4**).

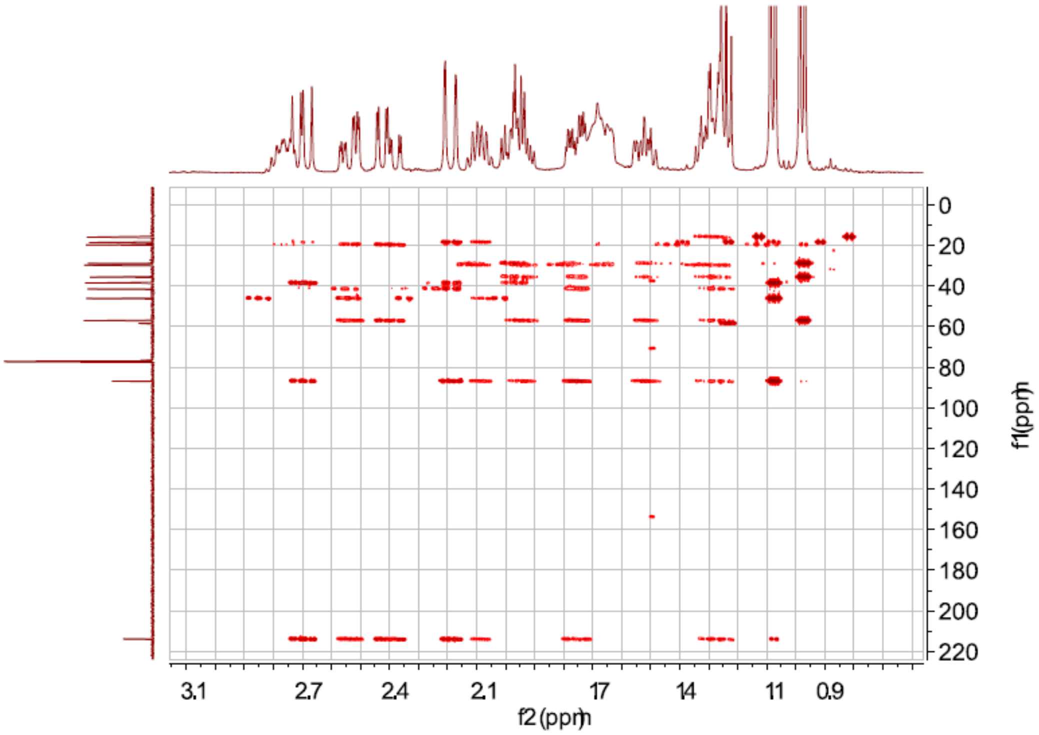

Figure S32. HMBC spectrum of compound (**4**).

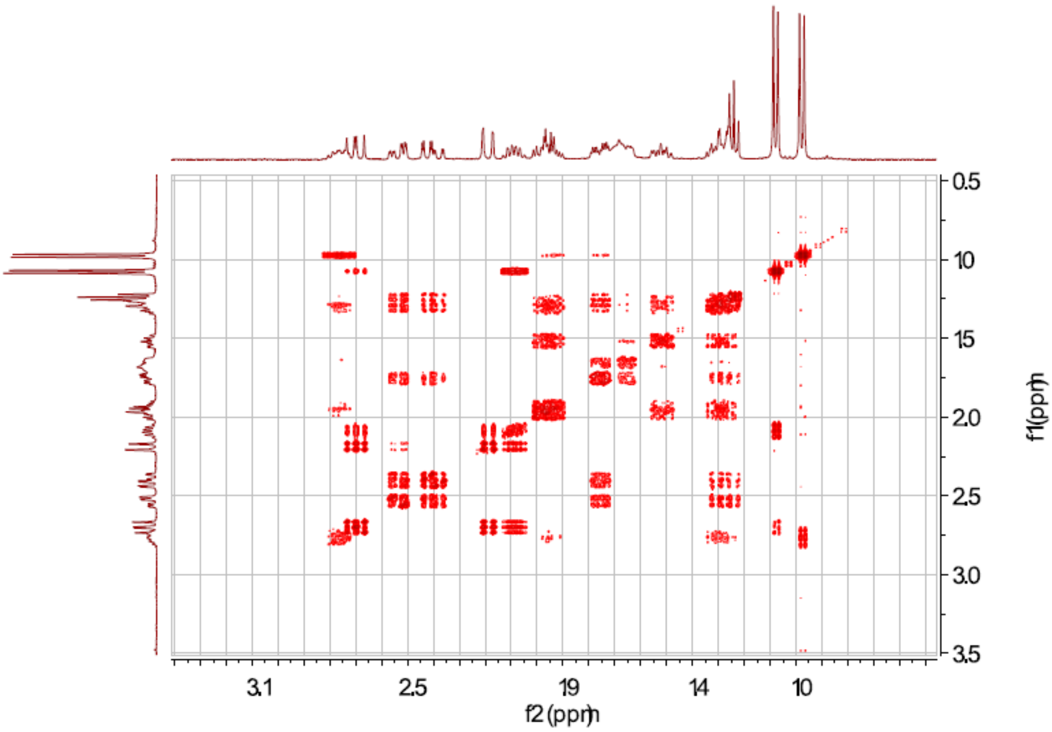

Figure S33. ¹H-¹H COSY spectrum of compound (**4**).

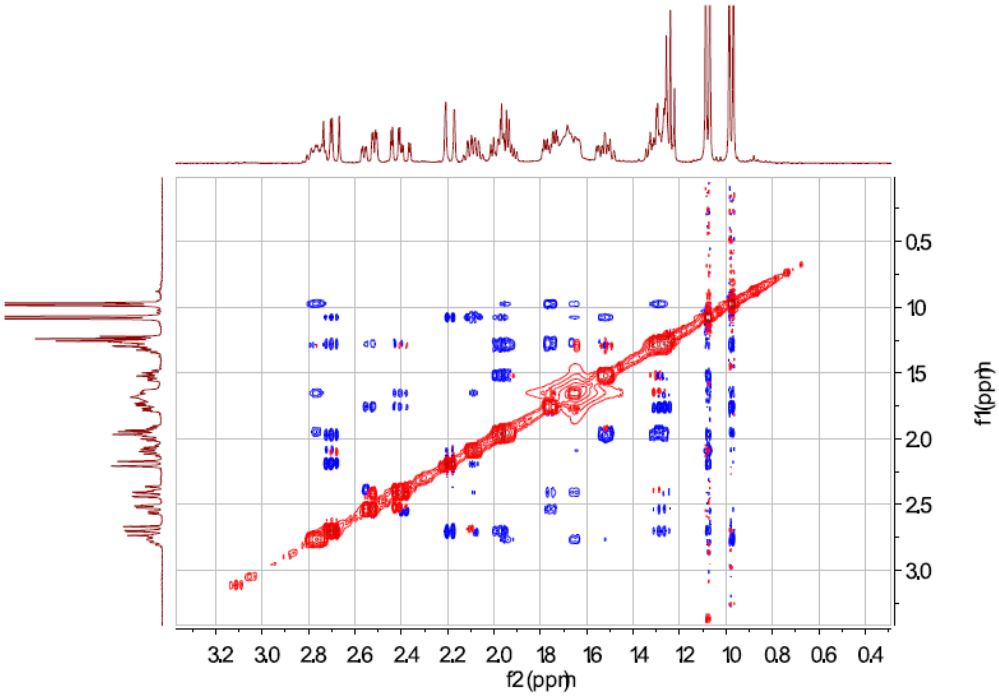

Figure S34. NOESY spectrum of compound (**4**) ($CDCl_3$, 400 MHz).

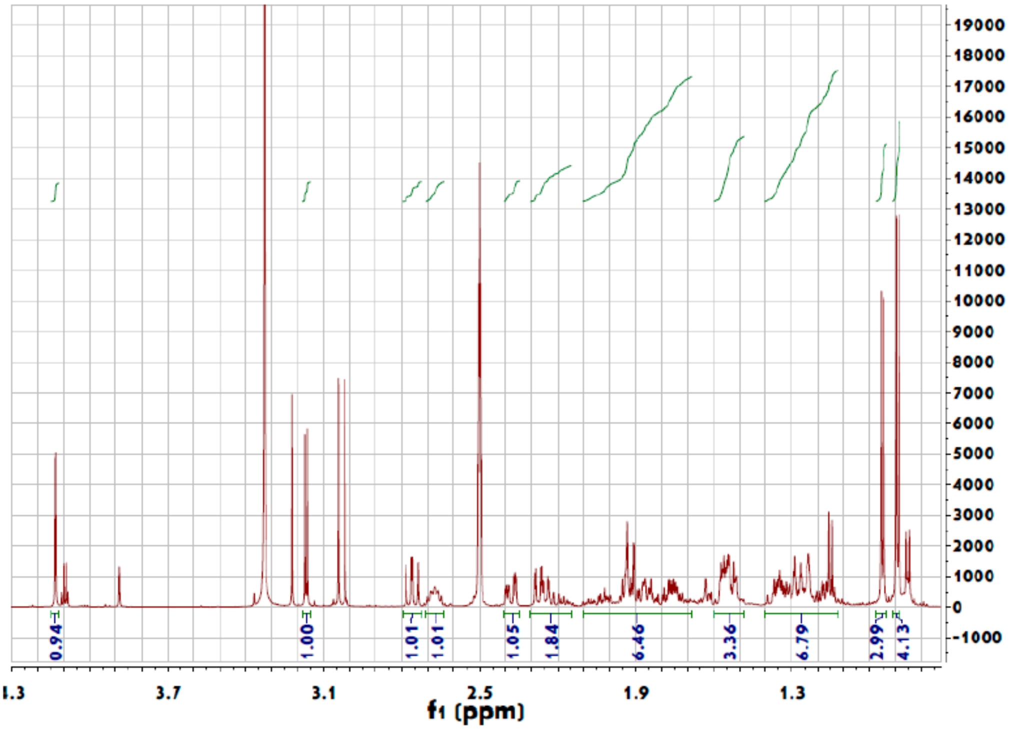

Figure S35.¹H NMR spectrum of compound (**4**) (DMSO-d6, 400 MHz).

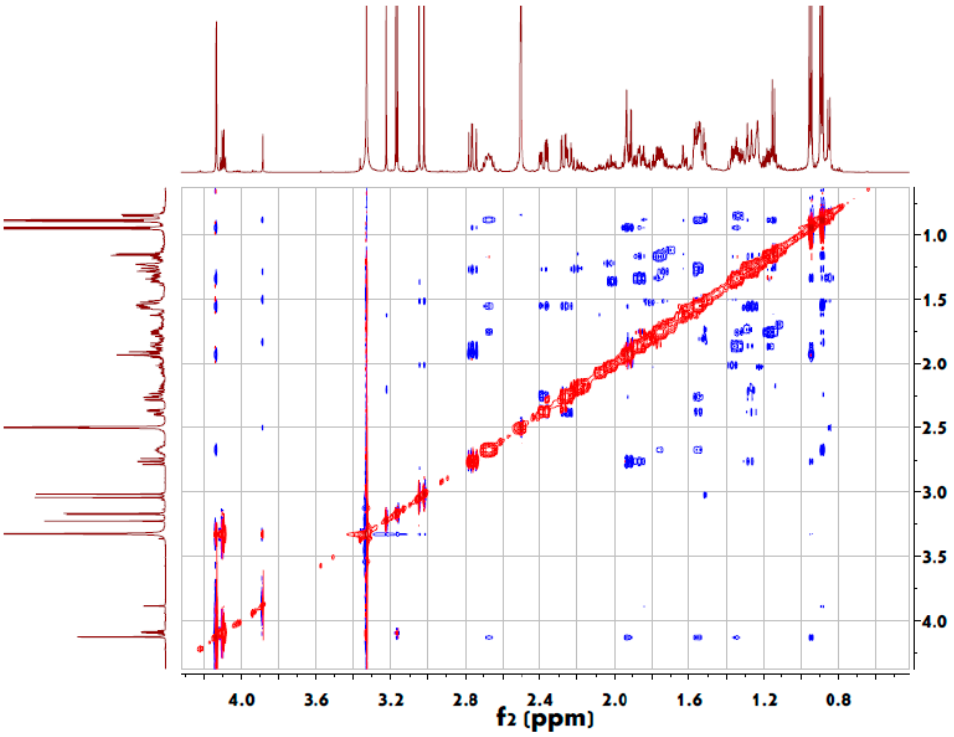

Figure S36. NOESY spectrum of compound (**4**) (DMSO-d6).

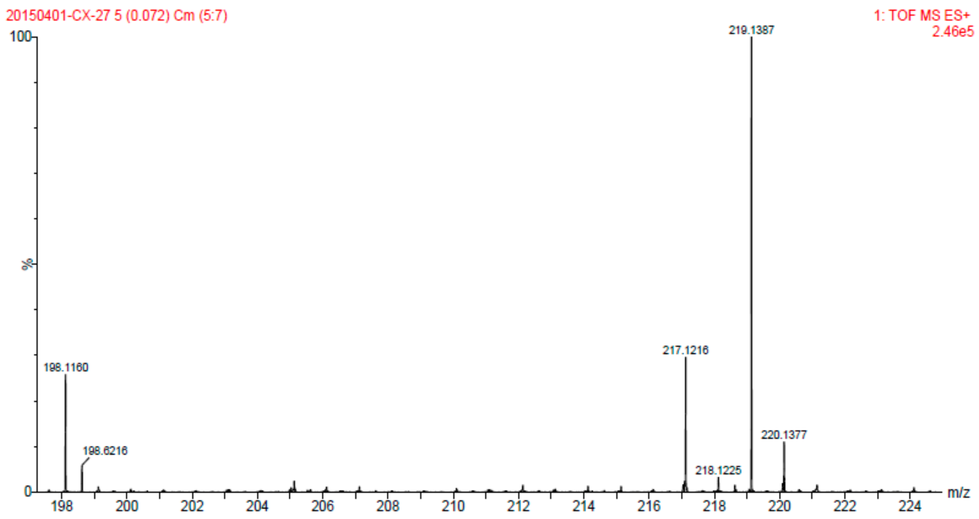

Figure S37. HR-ESIMS spectrum of compound (**4**).

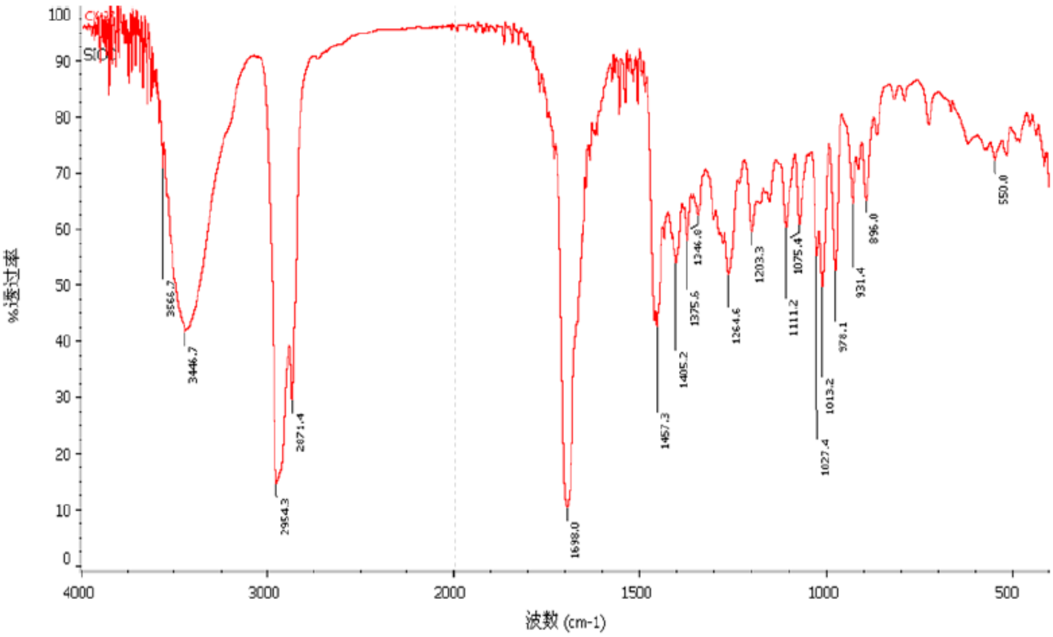

Figure S38. IR of compound (**4**).
